# Supplementary material for: Dynorphin Acts as a Neuromodulator to Inhibit Itch in the Dorsal Horn of the Spinal Cord
Source: Neuron. 2014 May 7;82(3):573–86. doi: 10.1016/j.neuron.2014.02.046 (PMC4022838; doi:10.1016/j.neuron.2014.02.046)
Supplement: Document S1. Supplemental Experimental Procedures, Figures S1–S5, and Table S1 [file mmc1.pdf]

Neuron, Volume *82*

Supplemental Information

Dynorphin Acts as a Neuromodulator to Inhibit Itch in the Dorsal Horn of the Spinal Cord

Adam P. Kardon, Erika Polgár, Junichi Hachisuka, Lindsey M. Snyder, Darren Cameron, Sinead Savage, Xiaoyun Cai, Sergei Karnup, Christopher R. Fan, Gregory M. Hemenway, Carcha S. Bernard, Erica S. Schwartz, Hiroshi Nagase, Christoph Schwarzer, Masahiko Watanabe, Takahiro Furuta, Takeshi Kaneko, H. Richard Koerber, Andrew J. Todd, and Sarah E. Ross

Supplemental Information

Dynorphin is a neuromodulator that inhibits itch in the dorsal horn of the spinal cord

Adam P. Kardon, Erika Polgár, Junichi Hachisuka, Lindsey M. Snyder, Darren Cameron, Sinead Savage, Xiaoyun Cai, Sergei Karnup, Christopher R. Fan, Gregory M. Hemenway, Carcha S. Bernard, Erica S. Schwartz, Hiroshi Nagase, Christoph Schwarzer, Masahiko Watanabe, Takahiro Furuta, Takeshi Kaneko, H. Richard Koerber, Andrew J. Todd, and Sarah E. Ross

Supplemental Data

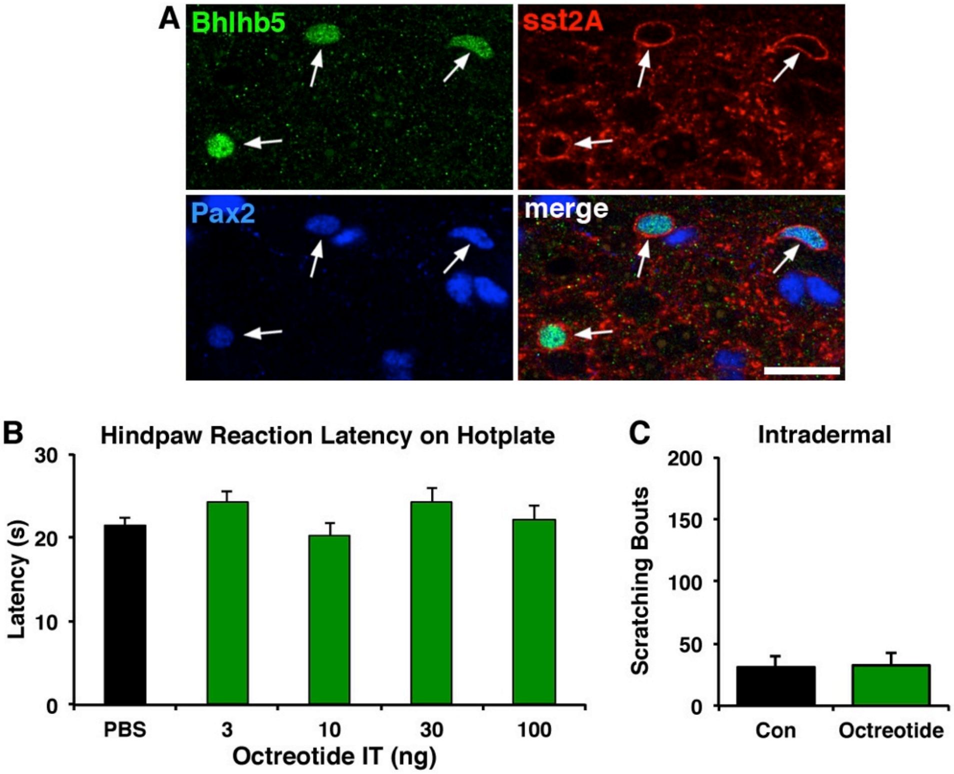

Figure S1, related to Figure 1. Analysis of sst_{2A}-expressing cells and the effect of the sst_{2A} agonist octreotide
A) B5-I neurons co-express sst_{2A} Bhlhb5-expressing neurons in the superficial dorsal horn that express Pax2 (B5-I neurons) also co-express sst_{2A}. Spinal cord sections from mice at post-natal day 4 (P4) were immunostained with antibodies directed against Bhlhb5 (green), sst_{2A} (red) and the inhibitory marker Pax2 (blue), as indicated. The vast majority (~90%) of cells expressing Bhlhb5 and Pax2 in laminae I and II co-label with sst_{2A} (arrows). A single confocal optical section of neurons in laminae I – IIo is shown. Scale bar = 20 µm**. B) Octreotide has no significant effect on acute thermal nociception when injected intrathecally** Response latency on a hotplate as assessed by lifting of the hindpaw or jumping following intrathecal injections of octreotide (3, 10, 30, or 100 ng) or vehicle (PBS). There was no significant difference between mice that received any dose of octreotide or vehicle. Two trials were averaged for each mouse (n = 6 – 8 mice/treatment). A one-way ANOVA was used to compare latency to react across treatments. **C) Octreotide does not elicit scratching behavior when injected intradermally.** Mice (n = 6 mice/treatment) received an intradermal injection of octreotide (100 ng) or vehicle (Con, 0.1X PBS) delivered into the nape of the neck, and were videotaped for forty minutes immediately following the injection. The number of scratch bouts by mice receiving intradermal octreotide was not significantly different than for those receiving vehicle alone. A Student's t-test was used to determine significance between treatment groups.

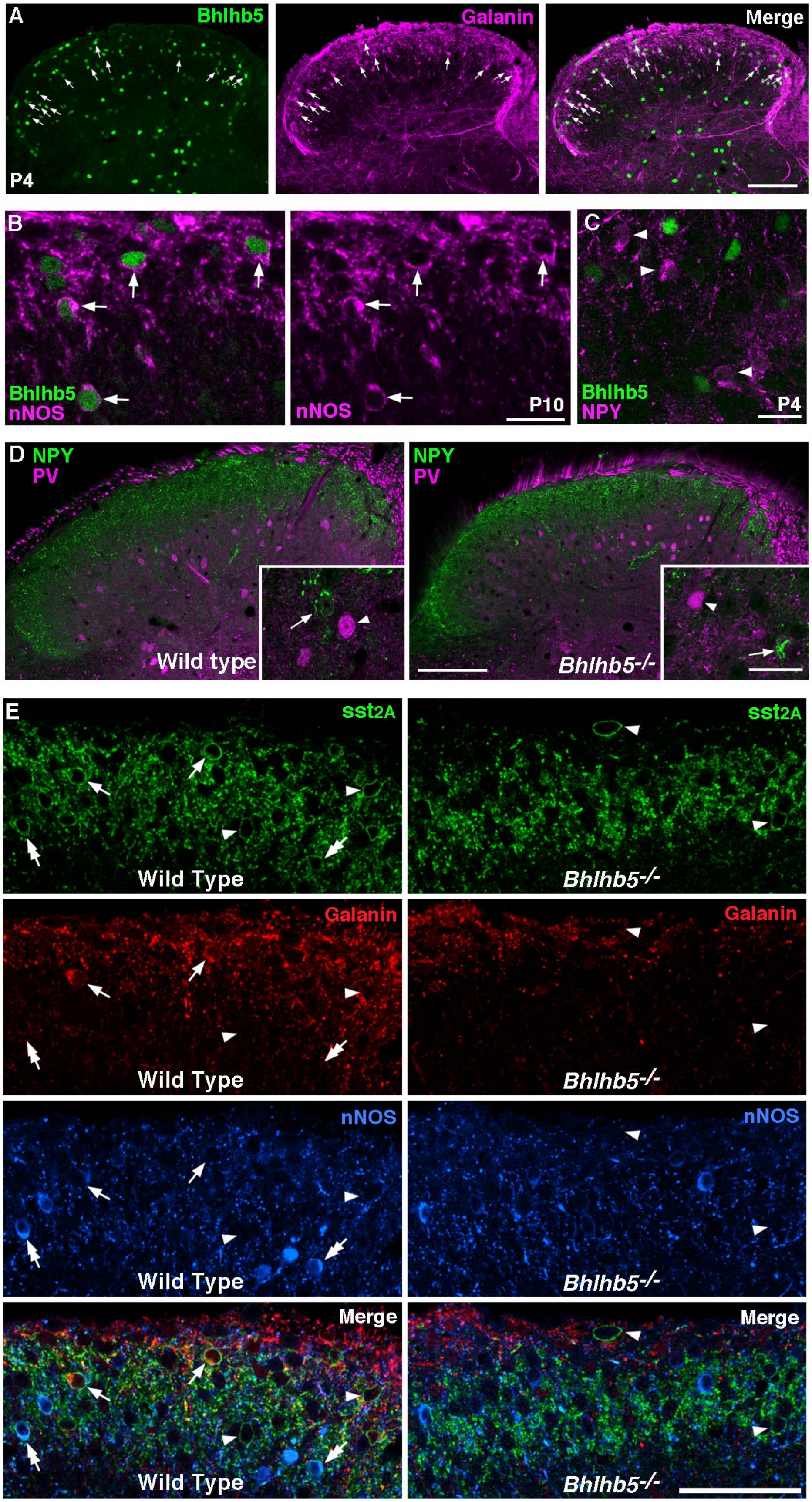

Figure S2, related to Figure 2. Immunohistochemical characterization of Bhlhb5-expressing interneurons in the dorsal horn of the spinal cord and comparison to *Bhlhb5* knockout mice

A) The majority of galanin-expressing neurons in the superficial dorsal horn of the spinal cord co-express Bhlhb5. Sections from P4 mice were immunostained with antibodies against Bhlhb5 (green) and galanin (magenta). A low magnification view (single confocal optical image) shows the entire dorsal horn. Merged view shows that virtually all galanin-positive neurons also express Bhlhb5 (arrows). Scale bar = 100 µm **B)** Large-field view of the section illustrated in Figure 2A from a P10 mouse reveals that many nNOS-expressing neurons are also Bhlhb5-immunoreative (arrows) Scale bar = 20 µm. **C)** Representative section from a P4 mouse immunostained for the presence of Bhlhb5 (green) and NPY (magenta) shows a lack of Bhlhb5 in NPY-expressing neurons (arrowheads). **D)** No change in the staining pattern for NPY or parvalbumin (PV) was observed in *Bhlhb5-/-* mice compared to wild type controls (sections from 6 mice examined for each genotype) Scale bar = 100 µm. Insets show individual NPY (arrows) and parvalbumin (arrowheads) cell bodies at higher magnification. Scale bar = 20µm. **E)** Larger field view of the section illustrated in Figure 2C, showing triple immunostaining with antibodies against sst_{2A} (green), galanin (red) and nNOS (blue) in 4 - 5 week old *Bhlhb5-/-* and wild type mice (n = 6 mice/genotype). Representative images demonstrate loss of the galanin- and nNOS-expressing subgroups (single- and double-headed arrows, respectively) of the sst_{2A}-immunoreactive population. The number of cells that express sst_{2A} but not galanin or nNOS (arrowheads) did not differ between *Bhlhb5-/-* and wild type mice. Scale bar = 50 µm.

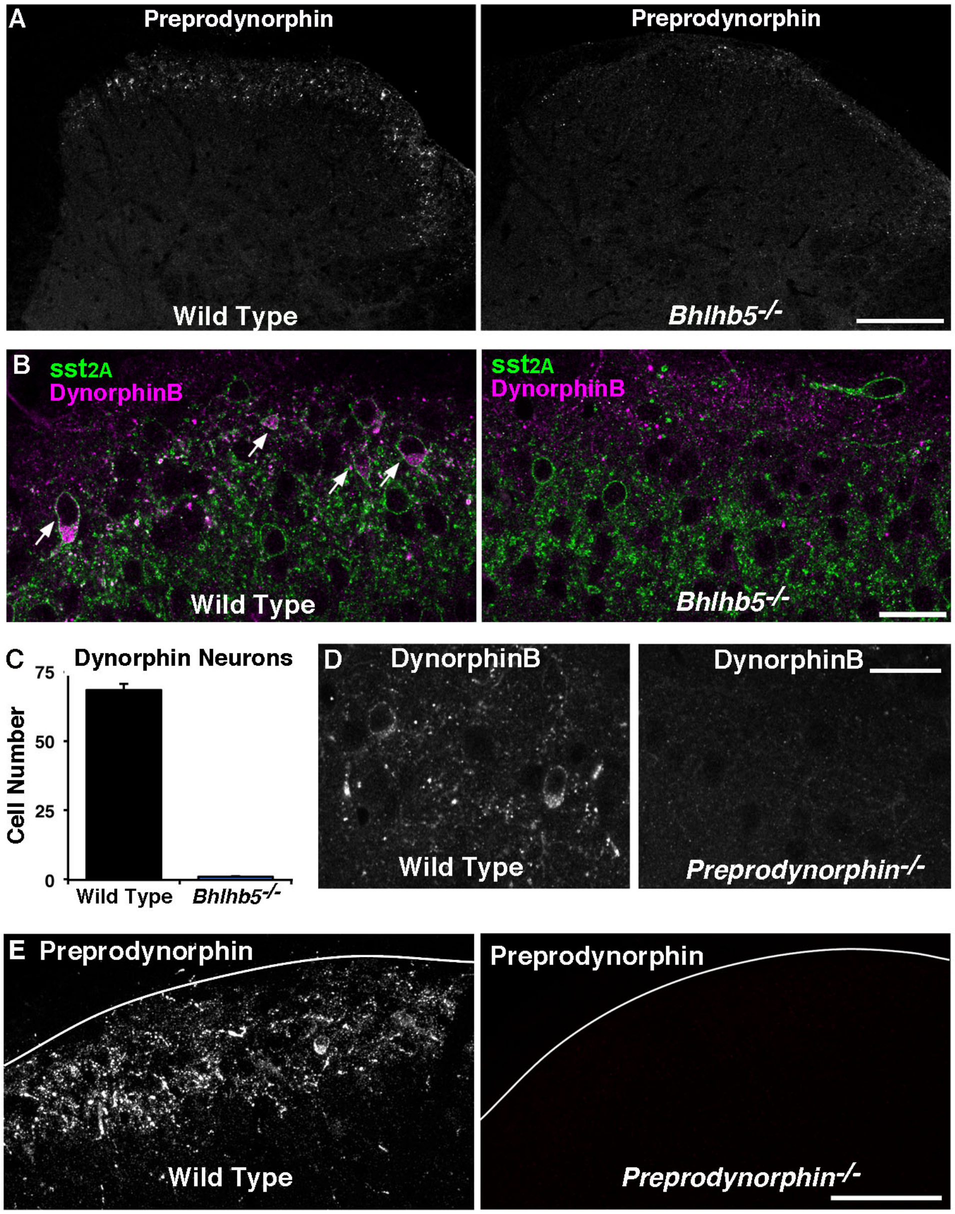

Figure S3, related to Figure 3. The number of dynorphin-expressing neurons is dramatically reduced in *Bhlhb5-/-* mice
A) Comparison of preprodynorphin immunoreactivity in 4 - 5 week old wild type and *Bhlhb5-/-* littermate pairs (representative confocal images, n = 3 mice/genotype). A low magnification view (single confocal optical image) reveals a dramatic loss of preprodynorphin-expressing neurons in laminae I-II. Scale bar = 100 µm. **B)** Similar results are obtained with an antibody against the dynorphin B peptide. Sections from wild type and *Bhlhb5-/-* mice were immunostained with antibodies directed against sst_{2A} (green) or dynorphin B (magenta). Dynorphin B-expressing sst_{2A}^{+} neurons in the wild type are indicated (arrows), but these are virtually absent in the *Bhlhb5-/-* mice. Scale bar = 20 µm. **C)** Quantification of (B). Data are mean + SEM number of cells in laminae I-II per dorsal horn through 100 µm cord taken from L4 (n = 3 mice/genotype). Note that the apparent number of dynorphin-expressing neurons as assessed with the dynorphin B antibody shown here is slightly lower than what is observed using the preprodynophin antibody (Figure 3C). This small difference is likely because the preprodynorphin antibody is more sensitive than the dynorphin B antibody, and can therefore detect cells that express very low levels of dynorphin. **D-E)** Immunostaining of tissue from wild type and *Preprodynorphin-/-* mice reveals that antibodies to dynorphin B (D) and preprodynorphin (E) are specific. Scale bar = 20 µm for D and 100 µm for E.

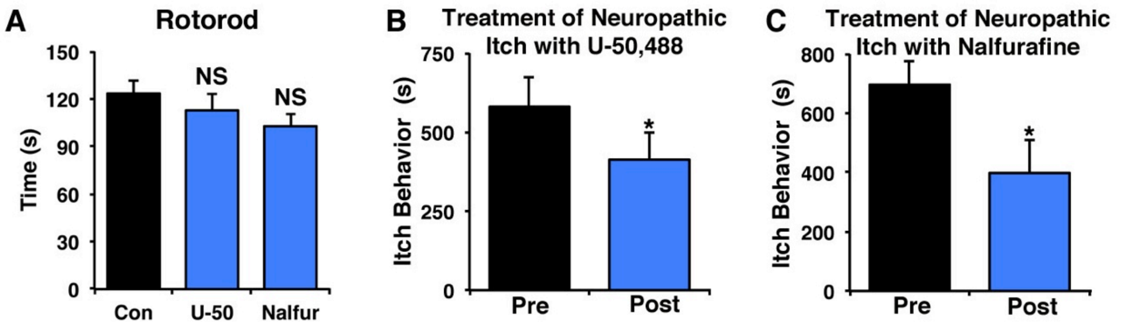

Figure S4, related to Figure 4. Effect of kappa agonists on motor function and neuropathic itch
A) No significant difference (NS, $p > 0.05$) in motor function is observed in a rotarod test upon treatment with U-50,488 (3 mg/kg) or nalfurafine (20 µg/kg) relative to control (PBS). Data are mean + SEM and analyzed using a one-way ANOVA ($n = 8$ mice/treatment). **B-C)** At 4 - 6 weeks of age, *Bhlhb5-/-* mice develop pruritic skin lesions, and can be considered a model of neuropathic itch. In order to assess the effect of kappa opioid receptor agonists on neuropathic itch, we measured the amount of time that *Bhlhb5-/-* mice with skin lesions spent biting/licking the affected site pre- and post-treatment. Mice with skin lesions spent the majority of the 30-minute observation period biting/licking the site of the lesion. However, following treatment with either (B) U-50,488 (3 mg/kg) or (C) nalfurafine (20 µg/kg), itch behavior is significantly reduced. Data are represented as mean + SEM ($n = 7$ - 11 mice/treatment). Statistical significance (*, $p < 0.05$) was determined by using a repeated measures t-test.

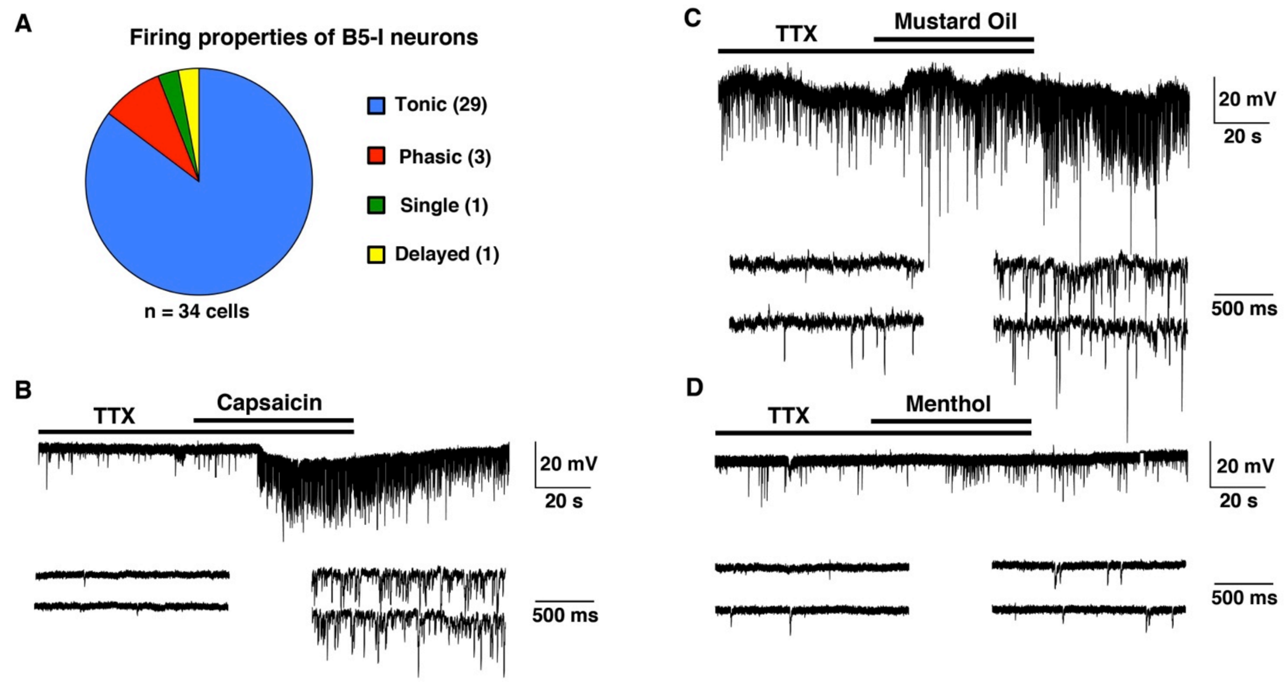

Figure S5, related to Figure 7. Electrophysiological analysis of B5-I neurons
A) The majority (29 of 34; 85%) of B5-I neurons showed tonic firing pattern upon injection of depolarising current. **B-D)** B5-I neurons receive direct input from TrpV1-, TrpA1- and TrpM8- expressing primary afferents. Increased mEPSC frequency following bath application of 2 µM capsaicin ($n = 3$, B), 100 µM AITC (mustard oil; $n = 5$, C) or 500 µM menthol ($n = 3$, D) was still observed in the presence of TTX (0.5 µM) to block action potential propagation, indicating that these effects are direct.

Supplemental Experimental Procedures:

Animal husbandry

Bhlhb5-/- mice were generated as previously described and are maintained on a mixed C57bl/6.129J background (Ross et al., 2010). To generate wild-type and *Bhlhb5-/-* mice for behavioral and immunohistochemical analyses, heterozygous *Bhlhb5-/+* mice were harem mated and age-matched wild-type and *Bhlhb5-/-* offspring from the resulting litters were used for subsequent experiments. Unless otherwise stated, behavioral experiments that involved *Bhlhb5-/-* mice were performed on 4 – 5 week old mice that did not have skin lesions. For electrophysiological experiments, *Bhlhb5-cre* mice, described in (Ross et al., 2010) were mated with Ai9 cre-responsive tdTomato reporter mice (The Jackson Laboratory; strain 007905). Mice were given free access to food and water and housed under standard laboratory conditions. The use of animals was approved by the Institutional Animal Care and Use Committee of the University of Pittsburgh and/or the Ethical Review Process Applications Panel of the University of Glasgow. Experiments performed in A.J.T.'s lab were in accordance with the UK Animals (Scientific Procedures) Act 1986.

Immunohistochemistry

Young adult or neonatal mice were deeply anaesthetized and fixed by perfusion with 4% freshly depolymerized formaldehyde. Transverse 60 µm thick sections were cut with a vibrating microtome from mid-lumbar spinal cord segments (L3 or L4) and processed free-floating for immunocytochemistry. Details of the primary antibodies used in this study are given in Supplementary Table 1. These were revealed with species-specific secondary antibodies raised in donkey and conjugated to Pacific Blue or Alexa 488 (both from Life Technologies) or to Rhodamine Red or DyLight 649 (both from Jackson Immunoresearch). In some cases tyramide signal amplification (TSA) was used. Sections were scanned with a confocal microscope (Zeiss LSM 710, with Argon multi-line, 405 nm diode, 561 nm solid state and 633 nm HeNe lasers lasers, and spectral detection system) through a 40× oil-immersion lens (NA 1.3) with the pinhole set to 1 Airy unit. Scans were analyzed with Neurolucida for Confocal (Microbrightfield). All quantitative analyses were carried out on the superficial dorsal horn (laminae I and II). In all cases involving comparison of knockout and wild-type mice, the observer was blind to the genotype.

Antibody characterization

The Bhlhb5 antibody was generated against the N-terminal part of the mouse protein, and detects a band at 37 kDa on Western blots of wild-type mouse brain that is absent in extracts from Bhlhb5 knockouts (Ross et al., 2010). The mouse monoclonal antibody NeuN, raised against cell nuclei from mouse brain, reacts with a neuron-specific protein (Mullen et al., 1992). In rat spinal cord, NeuN apparently detects all neurons, but does not label glial cells (Todd et al., 1998). Staining with the galanin antibody is absent from brains of galanin knockout mice (Makwana et al., 2010), and we have shown that staining in the dorsal horn with both galanin and NPY antibodies is abolished by preabsorption with the corresponding peptide (Rowan et al., 1993; Simmons et al., 1995). The two PPD antibodies were raised against the C-terminal 20 amino acids (FKVVTRSQENPNTYSEDLDV) of rat PPD (Lee et al., 1997), while the dynorphin B antibody was against the full peptide (YGGFLRRQFKVVT), which shows limited overlap with the sequence used to raise the PPD antibodies (Griffond et al., 1993). Staining with all 3 antibodies was absent in spinal cord sections from PPD knockout mice (Loacker et al., 2007). The Pax2 antibody was raised against amino acids 188-385 of the mouse protein, and recognizes bands of the appropriate size on Western blots of mouse embryonic kidney (Dressler and Douglass, 1992). The nNOS antibody was raised against recombinant rat protein and detects a band of 155 kDa in extracts of rat hypothalamus (Herbison et al., 1996). The sst_{2A} antibody was generated against the C terminal 15 amino acids of the mouse receptor, and staining is abolished by incubation with the immunizing peptide (manufacturer's specification). The parvalbumin antibody was raised against the mouse protein and recognizes a band at 13 kDa on Western blots of mouse brain (Nakamura et al., 2004).

Expression of neurochemical markers in wild type and Bhlhb5-/- mice

Sections from P4 or P10 C57bl/6J mice (University of Glasgow, Biological Services) were reacted with antibodies against Bhlhb5 and either anti-NPY, anti-galanin, or anti nNOS. Six *Bhlhb5-/-* and wild-type littermate pairs (4-5 week old) were used to determine the proportion of neurons that expressed sst_{2A}, galanin or nNOS. Sections were reacted with antibodies against each of these. Confocal image stacks (1 µm z-separation) were obtained from one dorsal horn from each animal and analyzed using a modification of the optical dissector method (Polgár et al., 2004). To examine the distribution of NPY and parvalbumin, 2 sections from each mouse were reacted with the corresponding antibodies and scanned with the confocal microscope. To look for alterations in the number of dynorphin-expressing cells, sections were reacted with sst_{2A} and either anti-PPD or anti-dynorphin B. One dorsal horn from each of 3 wild type and 3 *Bhlhb5* knockout mice were analyzed for each antibody combination. In order to ensure specificity of the PPD and dynorphinB antibodies, sections from wild-type and *preprodynorphin-/-* mice (Loacker et al., 2007) were reacted with these antibodies.

Identification of B5-I neurons in neonatal mice

Sections from three P4 C57bl/6J mice (University of Glasgow Biological Services) were reacted with cocktails of antibodies against Bhlhb5, NeuN, NPY, galanin, PPD, and/or Pax2, as indicated. To estimate the proportion of Bhlhb5-immunoreactive neurons in superficial dorsal horn that were inhibitory ($Pax2^+$), all $Bhlhb5^+/NeuN^+$ profiles in the dorsal horn on one side from each of two sections per animal were identified and plotted. These were then examined to determine whether they were Pax2-immunoreactive. Expression of galanin, NPY and PPD in $Bhlhb5^+$ neurons were assessed by determining the proportion of the neurons immunoreactive for each peptide that were also Bhlhb5-immunoreactive. In the case of galanin and PPD, we also

determined the proportion of Bhlhb5-expressing inhibitory interneurons (Bhlhb5$^+$/Pax2$^+$ cells) that were immunoreactive for these peptides. Since there was relatively little expression of nNOS in laminae I-II at P4, we reacted sections from 3 P10 mice with antibodies against nNOS, Pax2 and Bhlhb5. Confocal scans were examined to test whether any nNOS-expressing inhibitory interneurons (nNOS$^+$/Pax2$^+$ cells) were Bhlhb5-immunoreactive.

Table S1. Primary antibodies used in this study

Antibody	Host	Supplier/reference	Catalog number	Dilution
Bhlhb5	Rat	Ross et al., 2010		1:1,000
NeuN	Mouse	Millipore	MAB377	1:500-1,000
Galanin	Rabbit	Bachem	T-4334	1:20,000 (TSA)<br>1:1,000
PPD	Guinea pig	Lee et al., 1997		1:5,000 (TSA)
PPD	Rabbit	Lee et al., 1997		1:10,000 (TSA)
NPY	Rabbit	Bachem	T-4070	1:1,000
Pax2	Rabbit	Life technologies	716000	1:1,000
nNOS	Sheep	Herbison et al., 1996		1:2,000
sst_{2A}	Guinea pig	Gramsch laboratories	SS-870	1:2,000
parvalbumin	Guinea pig	Nakamura et al., 2004		1:2,500
Dynorphin B	Rabbit	Griffond et al., 1993		1:1,000

TSA: detected with tyramide signal amplification

Expression of neurochemical markers in wild-type and Bhlhb5-/- mice

Six (4- to 5-week old) mice of each genotype were used to determine the proportion of neurons that expressed sst_{2A}, galanin or nNOS. Sections were reacted with antibodies against each of these, together with NeuN antibody. Following incubation with fluorescent secondary antibodies, cell nuclei were stained with Sytox Orange (Life Technologies). Confocal image stacks (1 µm z-separation) were obtained from one dorsal horn from each animal and analyzed using a modification of the optical disector method (Polgár et al., 2004). The 5th and 40th optical sections in the z-series were set as reference and look-up, and all sections between these two were examined. The color channels representing NeuN and Sytox Orange were initially switched on, and all neuronal nuclei that were present on the reference section or any of the subsequent sections in the series, and that had disappeared by the look-up section, were drawn onto an outline of the gray matter. The channels representing sst_{2A}, galanin and nNOS were then viewed and the presence or absence of each type of immunoreactivity was noted for each neuron in the disector sample. Since sections can undergo a variable degree of shrinkage during processing, we corrected for this as described previously (Polgár et al. 2005). Briefly, the thickness of the mounted section was determined by scanning from the top to the bottom surface, and this was divided by 60 µm (the initial thickness) to provide a correction factor.

Since we found that all sst_{2A}-expressing cells remained Pax2-immunoreactive in adult wild-type mice, and that around 50% of all Pax2-expressing neurons in these animals were sst_{2A}-immunoreactive, we concluded that Pax2 can be used as a marker for most (if not all) inhibitory interneurons in the superficial dorsal horn in the adult. In order to determine whether there was loss among inhibitory interneurons that lacked sst_{2A}, we performed immunofluorescence labeling on sections from wild-type and *Bhlhb5-/-* mice with sst_{2A}, Pax2 and NeuN antibodies and then stained nuclei with DAPI (Sigma). Confocal image stacks (1 µm z-separation) were scanned through 2 dorsal horns from each mouse. These were analyzed by using the optical disector method, with a 10 µm separation between reference and look-up sections, and the number of Pax2$^+$ cells with or without sst_{2A} immunoreactivity was determined. Again, a correction was made for tissue shrinkage. To examine the distribution of NPY and parvalbumin, 2 sections from each mouse were reacted with the corresponding antibodies and scanned with the confocal microscope. To look for alterations in the number of dynorphin-expressing cells, sections were reacted with sst_{2A} and either anti-PPD or anti-dynorphin B. One dorsal horn from each of 3 wild-type and 3 *Bhlhb5* knockout mice were analyzed for each antibody combination. The sections were scanned through their full thickness (1 µm z-separation) and the number of sst_{2A}-immunoreactive cells that were either PPD- or dynorphin B-positive was determined.

Behavioral assessment

Unless otherwise noted, 6 - 8 week-old male C57bl/6J mice were used for all behavioral tests. The injection sites were shaved at least twenty-four hours prior to the start of an experiment. Mice were placed in clear plastic containers (3.5" x 3.5" x 5") for observation and allowed to acclimate for 30 minutes prior to all behavioral assessments. All assays were videotaped, and subsequently scored by an experimenter blind to treatment and/or genotype.

Intrathecal injections

A modified version (Lee et al., 2007) of the original intrathecal injection method (Hylden and Wilcox, 1980) was used. Mice were anesthetized (2.0% for induction and 1.5% for maintenance) with isoflurane in a flow of O_2, placed in a prone position, and the hair on their back was clipped. The caudal paralumbar region, just cranial to the iliac crests, was securely held by the thumb and middle fingers of the left hand, and the index finger was placed on the tip of sixth lumbar (L6) spinous process, the highest point of the vertebral column. All intrathecal injections were delivered in a total volume of 5 µl using a 30-gauge needle attached to a luer-tip 25 µl Hamilton syringe. The needle was inserted into the tissue at a 45° angle. The angle of the needle was maintained until the needle went through the fifth intervertebral space (L5–L6) and "slipped in" causing a sudden lateral movement of the tail. Solution was injected at a rate of 1 µl/s. The needle was held in position for 10 s and removed slowly to avoid any outflow of the solution. Anesthesia was discontinued and the mice recovered from anesthesia within 5 min.

Octreotide-evoked behavior

Mice were injected intrathecally with octreotide (3, 10, 30 or 100 ng) or vehicle (PBS), as described above. Immediately after recovering from the injection, mice were videotaped for 30 minutes to assess spontaneous scratching behavior. In some instances, chloroquine (100 µg in 10 µl PBS) was then injected intradermally into the calf of octreotide- and vehicle-treated mice. Itch behavior was defined as the cumulative amount of time spent biting/licking the injection site.

Acute itch behavior

5-HT (30 µg), histamine (100 µg), chloroquine (200 µg) and SLIGRL-NH2 (100 nM) were all dissolved in PBS and injected intradermally in a total volume of 20 µl into the nape of the neck. Each pruritogen evoked a robust scratching response that lasted approximately 30 minutes. Mice were pretreated systemically with U-50,488 (3 mg/kg; IP), nalfurafine (20 µg/kg; IP) or vehicle (50 µl PBS; IP) 30 minutes prior to the intradermal injections of pruritogens. The number of scratch bouts was counted in five-minute intervals over a forty-minute observation period.

AEW itch To model dry skin-mediated itch, spontaneous scratching behavior was induced by treatment twice daily with an acetone/diethylether (1:1) solution for 15 seconds followed by water for 30 seconds (AEW). Following five days of AEW treatment, mice received U-50,488, nalfurafine or vehicle and were videotaped for one hour. Scratch bouts directed to the nape of the neck and rostral back were counted in five-minute intervals.

Motor coordination

Mice were trained on the rotarod at a constant speed of 16 rpm until they could remain on for one minute without falling. On test day, mice were pretreated intraperitoneally with either U-50,488 (3mg/kg), nalfurafine (20µg/kg) or PBS. Each mouse performed three trials on the rotarod, which was accelerated from 4 rpm to 40 rpm over a period of five minutes. Trials were separated by 5-minute intervals for rest, and the time to fall off the apparatus was averaged across the trials for each mouse.

Cheek model of itch

For the cheek model of itch (Shimada and LaMotte, 2008), mice received an intradermal injection of either chloroquine (100 µg in 10 µl of PBS) or capsaicin (10 µl of 1% capsaicin, dissolved in a saline solution containing 7% Tween 80 and 20% ethanol) into the skin of the cheek. Observation chambers were surrounded by four mirrors such that the experimenter had an unobstructed view of the mouse's cheek. Scratch bouts with the hindlimb and instances of forelimb wiping were videotaped and quantified over a 40-minute observation period.

Calf model of itch

In some experiments, mice were pretreated with nalfurafine (40 ng), U-50,488 (10 µg) or vehicle (PBS) delivered intrathecally 30 min in advance. In other experiments, mice were pretreated with kappa antagonists 5'GNTI (1 µg), nor-BNI (1 µg) or vehicle, delivered intrathecally 24 h in advance, based on Munro et al., (2012). At the time of the experiment, chloroquine (100 µg in 10 µl PBS) was injected intradermally in the front of the calf, and the response was videotaped for 60 minutes. During real-time playback, biting is difficult to distinguish from other behaviors such as licking or grooming with the forepaws. However, when played at ¼ speed, biting could be identified as a gnawing motion in which the head moves at a frequency of approximately 15 Hz interspersed with abrupt head jerks, whereas licking was observed as a slow bobbing of the head at a frequency of approximately 4 Hz (LaMotte et al., 2011).

Inhibition of itch by menthol

Menthol (8%; Stopain, Troy Healthcare) or PBS (control) was applied topically in a volume of 10 µl to the cheek. Application of menthol resulted in modest wiping behavior in both wild-type and *Bhlhb5-/-* mice that ceased within ~ 5 min. Ten minutes after

the initial application of menthol or PBS, mice were given an intradermal injection of chloroquine (100 µg in 10 µl) into the cheek. Mice were videotaped and scratch bouts with the hindlimb were quantified over a 30-minute observation period.

Hotplate
Mice were lightly anesthetized with isoflurane, and injected intrathecally with octreotide (3, 10, 30 or 100 ng) or vehicle (PBS) in a total volume of 5 µl. One hour later, mice were placed directly on a hot plate set to 55°C. The response latency to hind paw licking or jumping was recorded over two trials for each mouse. Mice that did not respond at 45 seconds were removed from the hot plate to avoid tissue damage.

Electrophysiology
Four- to six-week old mice were deeply anesthetized with urethane (1.2 – 1.5 g/kg, i.p.). Thoracolumbar laminectomy was performed, and the thoracic and lumbar spinal cord was excised and placed it into an ice-cold, sucrose-based Krebs solution equilibrated with 95% O_2/5% CO_2. The composition of sucrose-based Krebs solution was as follows (mM); 234 sucrose, 2.5 KCl, 0.5 $CaCl_2$, 10 $MgSO_4$, 1.25 NaH_2PO_4, 26 $NaHCO_3$, 11 Glucose. Immediately after the removal of the spinal cord, the mice were killed by exsanguination. Dura and pia-arachnoid membrane were removed after cutting all of the ventral and dorsal roots. The spinal cord was mounted on a vibratome and parasagittal or transverse slices (250-300 µm thickness) were made. The slices were incubated in oxygenated, sucrose-based Krebs solution for at least 30 min in room temperature before recording. The slice was then transferred to a recording chamber. The slice was fixed on a nylon harp and perfused with Krebs solution saturated with 95% O_2 and 5% CO_2 at 36 ± 1 °C at flow rate of 10 ml/min. The Krebs solution contained (mM): 117 NaCl, 3.6 KCl, 2.5 $CaCl_2$, 1.2 $MgCl_2$, 1.2 NaH_2PO_4, 25 $NaHCO_3$ and 11 glucose.

Lamina II was identified as a translucent band in the dorsal horn. Individual neurons were identified with a 40x objective lens under IR-DIC optics (BX51WI Olympus microscope). The microscope was coupled with a CCD camera (ORCA-ER Hamamatsu Photonics) and monitor screen. Genetically labeled *Bhlhb5-cre* neurons in lamina II of the dorsal horn were identified by their fluorescence. B5-I neurons were distinguished within this subset by hyperpolarization upon bath application of 1 µM somatostatin. To determine firing pattern of B5-I neurons, 1 s depolarizing current steps (20 – 200 pA in 10 pA steps) were applied. Because firing pattern can vary depending on holding potential, we tested the B5-I neurons at several different holding potentials. Firing patterns were determined in response to depolarizing current injections of 1 s duration from each of the potentials. Classifications were based on (Heinke et al., 2004; Ruscheweyh et al., 2004; Ruscheweyh and Sandkuhler, 2002).

Whole-cell patch-clamp recordings were made with patch-pipette electrodes with a resistance of 6-12 MΩ. The composition of the pipette solution was as follows (mM); 135 potassium gluconate, 5 KCl, 0.5 $CaCl_2$, 5 EGTA, 5 Hepes, 5 ATP-Mg, pH 7.2. Neurobiotin 0.2% or Alexa Fluor 488 25 µM were added for morphological experiments. The signals were acquired with an amplifier (Axopatch 200B, Molecular Devices, California). The data were digitized with an A/D converter (Digidata 1322A, Molecular Devices) and stored on a personal computer using a data acquisition program (Clampex version 9.0, Molecular Devices).

Cell recordings were made in voltage-clamp mode at holding potentials of -70mV to record excitatory postsynaptic currents (EPSCs). At this potential GABA-/ glycine-mediated inhibitory postsynaptic currents were negligible (Yoshimura and Nishi, 1993). Frequency and amplitude of EPSCs were analysed by using MiniAnalysis program (Synaptosoft, Inc.). We defined neurons as being sensitive to a particular drug when the frequency or amplitude of the synaptic responses was altered by more than ± 50 % of control. The drugs were dissolved in Krebs solution and applied by exchanging solutions via a three-way stopcock. The drugs used were somatostatin (1 µM, Sigma-Aldrich), capsaicin (2 *µ*M; Sigma-Aldrich), allyl isothiocyanate (100 µM; Sigma-Aldrich), menthol (500 µM; Sigma-Aldrich) and TTX (0.5 µM, Tocris).

Morphological reconstructions and characterization
Biocytin-filled cells were visualized as described previously (Karnup and Stelzer, 1999). Briefly, fixed slices were embedded in 10% gelatin and sectioned at 100 µm thickness using a vibratome (Leica Microsystems, Wetzlar, Germany). The sections were reacted with 1% H_2O_2, 0.5% Triton X-100, ABC complex, and Ni-DAB chromagen. After dehydration, the sections were mounted in DPX. Two-dimensional reconstructions of filled neurons were made with Neurolucida software (MicroBrightField, Colchester, VT).

Following reconstruction, neurons were classified into one of 5 major categories as previously identified (Grudt and Perl, 2002; Yasaka et al., 2007; Zheng et al., 2010) in the hamster, rat, and mouse, respectively. According to this scheme, islet cells display an elongated dentritic tree in the rostral-caudal dimension greater than 400 µm and a more limited dorsal-ventral and medial-lateral spread (<100 µm). The arborization of islet cells is mostly restricted to lamina II. Central cells show similar morphological characteristics to islet cells; however, the extent of the dendritic arbor in the rostral-caudal dimension is much less (~200 µm). Vertical cells show a greater dorsal-ventral spread of the dentritic tree into lamina III and sometimes lamina I (~200 µm). Radial cells display dendrites that spread in all directions at approximately equal lengths (between 50-150 µm) across all dimensions and remained mostly within lamina II. Cells not matching any of the above mentioned categories are designated as unclassified.

Supplemental References

Dressler, G.R., and Douglass, E.C. (1992). Pax-2 is a DNA-binding protein expressed in embryonic kidney and Wilms tumor. Proceedings of the National Academy of Sciences U S A *89*, 1179-1183.

Griffond, B., Deray, A., Fellmann, D., Ciofi, P., Croix, D., and Bugnon, C. (1993). Colocalization of prolactin- and dynorphin-like substances in a neuronal population of the rat lateral hypothalamus. Neuroscience Letters *156*, 91-95.

Grudt, T.J., and Perl, E.R. (2002). Correlations between neuronal morphology and electrophysiological features in the rodent superficial dorsal horn. The Journal of physiology *540*, 189-207.

Heinke, B., Ruscheweyh, R., Forsthuber, L., Wunderbaldinger, G., and Sandkuhler, J. (2004). Physiological, neurochemical and morphological properties of a subgroup of GABAergic spinal lamina II neurones identified by expression of green fluorescent protein in mice. The Journal of physiology *560*, 249-266.

Herbison, A.E., Simonian, S.X., Norris, P.J., and Emson, P.C. (1996). Relationship of neuronal nitric oxide synthase immunoreactivity to GnRH neurons in the ovariectomized and intact female rat. Journal of Neuroendocrinology *8*, 73-82.

Hylden, J.L., and Wilcox, G.L. (1980). Intrathecal morphine in mice: a new technique. European Journal of Pharmacology *67*, 313-316.

Karnup, S., and Stelzer, A. (1999). Temporal overlap of excitatory and inhibitory afferent input in guinea-pig CA1 pyramidal cells. The Journal of physiology *516 (Pt 2)*, 485-504.

LaMotte, R.H., Shimada, S.G., and Sikand, P. (2011). Mouse models of acute, chemical itch and pain in humans. Experimental dermatology *20*, 778-782.

Lee, T., Kaneko, T., Taki, K., and Mizuno, N. (1997). Preprodynorphin-, preproenkephalin-, and preprotachykinin-expressing neurons in the rat neostriatum: an analysis by immunocytochemistry and retrograde tracing. Journal of Comparative Neurology *386*, 229-244.

Lee, H., Naughton, N.N., Woods, J.H., and Ko, M.C. (2007). Effects of butorphanol on morphine-induced itch and analgesia in primates. Anesthesiology *107*, 478-485.

Loacker, S., Sayyah, M., Wittmann, W., Herzog, H., and Schwarzer, C. (2007). Endogenous dynorphin in epileptogenesis and epilepsy: anticonvulsant net effect via kappa opioid receptors. Brain : a journal of neurology *130*, 1017-1028.

Makwana, M., Werner, A., Acosta-Saltos, A., Gonitel, R., Pararajasingham, A., Ruff, C., Rumajogee, P., Cuthill, D., Galiano, M., Bohatschek, M.*, et al.* (2010). Peripheral facial nerve axotomy in mice causes sprouting of motor axons into perineuronal central white matter: time course and molecular characterization. Journal of Comparative Neurology *518*, 699-721.

Munro, T.A., Berry, L.M., Van't Veer, A., Beguin, C., Carroll, F.I., Zhao, Z., Carlezon, W.A., Jr., and Cohen, B.M. (2012). Long-acting kappa opioid antagonists nor-BNI, GNTI and JDTic: pharmacokinetics in mice and lipophilicity. BMC Pharmacology *12*, 5.

Mullen, R.J., Buck, C.R., and Smith, A.M. (1992). NeuN, a neuronal specific nuclear protein in vertebrates. Development *116*, 201-211.

Nakamura, M., Sato, K., Fukaya, M., Araishi, K., Aiba, A., Kano, M., and Watanabe, M. (2004). Signaling complex formation of phospholipase Cbeta4 with metabotropic glutamate receptor type 1alpha and 1,4,5-trisphosphate receptor at the perisynapse and endoplasmic reticulum in the mouse brain. The European journal of neuroscience *20*, 2929-2944.

Polgár, E., Gray, S., Riddell, J.S., and Todd, A.J. (2004). Lack of evidence for significant neuronal loss in laminae I-III of the spinal dorsal horn of the rat in the chronic constriction injury model. Pain *111*, 144-150.

Polgar, E., Hughes, D.I., Arham, A.Z., and Todd, A.J. (2005). Loss of neurons from laminas I-III of the spinal dorsal horn is not required for development of tactile allodynia in the spared nerve injury model of neuropathic pain. The Journal of neuroscience : the official journal of the Society for Neuroscience *25*, 6658-6666.

Ross, S.E., Mardinly, A.R., McCord, A.E., Zurawski, J., Cohen, S., Jung, C., Hu, L., Mok, S.I., Shah, A., Savner, E.M.*, et al.* (2010). Loss of inhibitory interneurons in the dorsal spinal cord and elevated itch in Bhlhb5 mutant mice. Neuron *65*, 886-898.

Rowan, S., Todd, A.J., and Spike, R.C. (1993). Evidence that neuropeptide Y is present in GABAergic neurons in the superficial dorsal horn of the rat spinal cord. Neuroscience *53*, 537-545.

Ruscheweyh, R., Ikeda, H., Heinke, B., and Sandkuhler, J. (2004). Distinctive membrane and discharge properties of rat spinal lamina I projection neurones in vitro. The Journal of physiology *555*, 527-543.

Ruscheweyh, R., and Sandkuhler, J. (2002). Lamina-specific membrane and discharge properties of rat spinal dorsal horn neurones in vitro. The Journal of physiology *541*, 231-244.

Shimada, S.G., and LaMotte, R.H. (2008). Behavioral differentiation between itch and pain in mouse. Pain *139*, 681-687.

Simmons, D.R., Spike, R.C., and Todd, A.J. (1995). Galanin is contained in GABAergic neurons in the rat spinal dorsal horn. Neuroscience Letters *187*, 119-122.

Todd, A.J., Spike, R.C., and Polgar, E. (1998). A quantitative study of neurons which express neurokinin-1 or somatostatin sst2a receptor in rat spinal dorsal horn. Neuroscience *85*, 459-473.

Yasaka, T., Kato, G., Furue, H., Rashid, M.H., Sonohata, M., Tamae, A., Murata, Y., Masuko, S., and Yoshimura, M. (2007). Cell-type-specific excitatory and inhibitory circuits involving primary afferents in the substantia gelatinosa of the rat spinal dorsal horn in vitro. The Journal of physiology *581*, 603-618.

Yoshimura, M., and Nishi, S. (1993). Blind patch-clamp recordings from substantia gelatinosa neurons in adult rat spinal cord slices: pharmacological properties of synaptic currents. Neuroscience *53*, 519-526.

Zheng, J., Lu, Y., and Perl, E.R. (2010). Inhibitory neurones of the spinal substantia gelatinosa mediate interaction of signals from primary afferents. The Journal of physiology *588*, 2065-2075.
