# Supplementary material for: Dynorphin Acts as a Neuromodulator to Inhibit Itch in the Dorsal Horn of the Spinal Cord
Source: Neuron. 2014 May 7;82(3):573–86. doi: 10.1016/j.neuron.2014.02.046 (PMC4022838; doi:10.1016/j.neuron.2014.02.046)
Supplement: Document S2. Article plus Supplemental Information [file mmc2.pdf]

Dynorphin Acts as a Neuromodulator to Inhibit Itch in the Dorsal Horn of the Spinal Cord

Adam P. Kardon,[1,2,9] Erika Polgár,[3,9] Junichi Hachisuka,[1,2] Lindsey M. Snyder,[1,2] Darren Cameron,[3] Sinead Savage,[3] Xiaoyun Cai,[1,2] Sergei Karnup,[1,2] Christopher R. Fan,[1] Gregory M. Hemenway,[1] Carcha S. Bernard,[1] Erica S. Schwartz,[2,4] Hiroshi Nagase,[5] Christoph Schwarzer,[6] Masahiko Watanabe,[7] Takahiro Furuta,[8] Takeshi Kaneko,[8] H. Richard Koerber,[1,2] Andrew J. Todd,[3,*] and Sarah E. Ross[1,2,4,*]
[1]Department of Neurobiology
[2]University of Pittsburgh Pain Center
University of Pittsburgh, 200 Lothrop St. Pittsburgh, PA 15213, USA
[3]Institute of Neuroscience and Psychology, College of Medical, Veterinary and Life Sciences, University of Glasgow, Glasgow G12 8QQ, UK
[4]Department of Anesthesiology, University of Pittsburgh, 200 Lothrop Street, Pittsburgh, PA 15213, USA
[5]International Institute for Integrative Sleep Medicine, University of Tsukuba, Life Science Center of Tsukuba Advanced Research Alliance C-1F, 1-1-1 Tenoudai Tsukuba Ibaraki, Tsukuba 305-8577, Japan
[6]Department of Pharmacology, Innsbruck Medical University, A-6020 Innsbruck, Austria
[7]Department of Anatomy, Hokkaido University School of Medicine, Sapporo 060-8638, Japan
[8]Department of Morphological Brain Science, Graduate School of Medicine, Kyoto University, Kyoto 606-8501, Japan
[9]Co-first authors
*Correspondence: andrew.todd@glasgow.ac.uk (A.J.T.), saross@pitt.edu (S.E.R.)
http://dx.doi.org/10.1016/j.neuron.2014.02.046
This is an open access article under the CC BY license (http://creativecommons.org/licenses/by/3.0/).

SUMMARY

Menthol and other counterstimuli relieve itch, resulting in an antipruritic state that persists for minutes to hours. However, the neural basis for this effect is unclear, and the underlying neuromodulatory mechanisms are unknown. Previous studies revealed that *Bhlhb5*$^{-/-}$ mice, which lack a specific population of spinal inhibitory interneurons (B5-I neurons), develop pathological itch. Here we characterize B5-I neurons and show that they belong to a neurochemically distinct subset. We provide cause-and-effect evidence that B5-I neurons inhibit itch and show that dynorphin, which is released from B5-I neurons, is a key neuromodulator of pruritus. Finally, we show that B5-I neurons are innervated by menthol-, capsaicin-, and mustard oil-responsive sensory neurons and are required for the inhibition of itch by menthol. These findings provide a cellular basis for the inhibition of itch by chemical counterstimuli and suggest that kappa opioids may be a broadly effective therapy for pathological itch.

INTRODUCTION

Itch, like pain, is an aversive sensation that warns us of potential threats to the body (Ross, 2011; Bautista et al., 2014). However, itch is a distinct sensation, characterized by the desire to scratch. Although scratching may remove irritants from the skin (providing at least transient relief from itch), it has the paradoxical effect of causing tissue damage that potentiates itch through release of inflammatory mediators. This pathological itch-scratch-itch cycle is a hallmark of chronic pruritus, which can be just as debilitating as chronic pain (Weisshaar and Dalgard, 2009; Yosipovitch, 2008). Unfortunately, there are few therapeutic options for those that suffer from severe pathological itch. Whereas mu opioids such as morphine are highly effective for the treatment of pain, these drugs actually worsen itch (Ko and Naughton, 2000; Szarvas et al., 2003). Thus, there is a great need for better therapies to treat intractable pruritus.

One reason that itch has lagged behind pain in terms of effective therapies is because, until recently, we lacked a clear understanding of how itch is detected and encoded in the nervous system. However, over the last few years there has been much progress in this field. There is now good evidence that MrgprA3-expressing sensory neurons selectively mediate itch, even when activated by the classic algogen capsaicin (Han et al., 2013). It is very likely that these are not the only itch-selective fibers, since histamine-dependent itch appears to be mediated by a different subset of sensory neurons (Roberson et al., 2013). Next, itch seems to be relayed by at least two populations of spinal interneurons—those that express the Npra receptor and those that express the gastrin-releasing peptide receptor (GRPR)—before being conveyed to the brain where it is consciously perceived (Mishra and Hoon, 2013; Sun et al., 2009).

Menthol and other forms of counterstimulation, such as scratching, heat, cool, and noxious agents, provide relief of itch that begins almost instantaneously and lasts from minutes to hours (Ward et al., 1996; Yosipovitch et al., 2007; Bromm et al., 1995). This relief occurs even when the counterstimulus is applied at great distances from the source of itch sensation (Nilsson et al., 1997). Together, these psychophysical observations suggest that crossmodal inhibition occurs centrally, possibly within the spinal dorsal horn, where sensory information is first integrated and modulated (Todd, 2010). The instantaneous relief of itch

experienced upon scratching is presumably mediated by a fast-acting neurotransmitter (Akiyama et al., 2011). In contrast, prolonged inhibition is thought to involve neuromodulators, but the nature of such neuromodulation remains elusive and the neural basis for inhibition of itch by counterstimuli is not known.

We previously generated a mouse model of pathological chronic itch through the constitutive deletion of *Bhlhb5* (also known as *Bhlhe22*), a transcription factor that is transiently expressed in several neuronal subtypes during embryonic and early postnatal development (Ross et al., 2010, 2012). Through selective ablation, we provided strong evidence that the pathological itch in *Bhlhb5* mutant mice was due to loss of Bhlhb5 in inhibitory neurons in the spinal dorsal horn. Using fate-mapping approaches, we found that *Bhlhb5* mutant mice lack a subset of inhibitory neurons in laminae I and II (Ross et al., 2010). These findings suggested that *Bhlhb5* is essential for the survival of a set of spinal inhibitory interneurons (termed B5-I neurons) that are required for normal itch sensation. However, the identity of B5-I neurons was not clear, and how they inhibit itch was not known.

Here we provide evidence that acute inhibition of B5-I neurons results in elevated itch. We identify and characterize B5-I neurons, showing that they correspond to specific neurochemically defined populations and that they release the kappa opioid dynorphin. Our data suggest that kappa agonists act locally within the spinal cord to selectively reduce itch and not pain. We find that B5-I cells are directly innervated by primary afferents that respond to counterstimuli, such as heat and coolness, which relieve itch in humans. Moreover, we show that menthol inhibits itch in wild-type mice but does not do so in mice lacking B5-I neurons. Thus, B5-I neurons may mediate the inhibition of itch by chemical counterstimuli.

RESULTS

Acute Inhibition of B5-I Cells Results in Elevated Itch

We previously showed that Bhlhb5 is needed for survival of spinal inhibitory interneurons that are required for normal itch sensation (Ross et al., 2010). To more specifically identify these neurons, we performed coimmunostaining for Bhlhb5 and markers that define distinct populations of spinal interneurons. Bhlhb5 is transiently expressed in ~7% of neurons in the dorsal horn of mice from embryonic day 13.5 to postnatal day 10 (P10), so we performed these experiments using P4 mice. Consistent with our previous report (Ross et al., 2010), we found that three-quarters of Bhlhb5-expressing neurons in superficial dorsal horn (laminae I and II) are inhibitory, as shown by coexpression of Pax2 (Figure 1A). We refer to these Bhlhb5-expressing inhibitory interneurons as *B5-I* neurons.

The somatostatin receptor sst_{2A} is exclusive to inhibitory neurons in superficial dorsal horn and is found in ~50% of the inhibitory interneurons in this region (Polgár et al., 2013a, 2013b; Todd et al., 1998; Yasaka et al., 2010). To determine whether the B5-I neurons belonged to this subset, we used antibodies against Bhlhb5, Pax2, and sst_{2A}. This immunostaining showed that the vast majority of B5-I neurons (~90%) coexpressed the somatostatin receptor sst_{2A} (Figures 1A and S1A available online). Furthermore, when we recorded from spinal interneurons genetically labeled with the *Bhlhb5-cre* allele (Ross et al., 2010), half showed strong hyperpolarization in response to somatostatin (Figure 1B), confirming that B5-I neurons express functional sst_{2A} receptors. Given the loss of B5-I neurons in *Bhlhb5*$^{-/-}$ mice, we reasoned that there would be a corresponding decrease in the number of sst_{2A}-expressing neurons in these animals. As predicted, the number of sst_{2A}-expressing neurons was reduced by two-thirds in *Bhlhb5*$^{-/-}$ mice, with no significant change in the number of sst_{2A}-negative inhibitory neurons (Figures 1C and 1D). Thus, the vast majority of B5-I neurons belong to the subset of inhibitory spinal interneurons that express sst_{2A}, and a large proportion of the sst_{2A}-expressing population is missing in *Bhlhb5*$^{-/-}$ mice.

Since somatostatin inhibits neuronal activity and sst_{2A} is the only somatostatin receptor that is expressed by dorsal horn neurons (see http://www.brain-map.org), the finding that B5-I neurons express sst_{2A} allowed us to directly test the idea that B5-I neurons normally function to inhibit itch. This experiment was important because, although we had previously shown that loss of B5-I neurons during development is associated with abnormally elevated itch (Ross et al., 2010), the evidence was merely correlative. Specifically, it was not clear whether B5-I neurons function in the adult to inhibit itch, or whether B5-I neurons play a key developmental role in the formation of proper itch circuits. We hypothesized that if B5-I neurons normally function to inhibit itch, then acute inhibition of these neurons by somatostatin would increase itch sensitivity (Figure 1E). Indeed, upon intrathecal injection of the somatostatin analog octreotide, we observed vigorous scratching, biting, and licking behavior that was suggestive of itch (Figure 1F), consistent with previous reports (Seybold et al., 1982). This spontaneous behavior was dose dependent, with an immediate onset and a duration of approximately half an hour.

Because B5-I neurons account for the majority (two-thirds) of sst_{2A}-expressing cells, the finding that acute treatment with octreotide results in elevated scratching behavior is consistent with the hypothesis that B5-I neurons inhibit itch. Nevertheless, it remained possible that the observed scratching behavior was due instead to the effect of octreotide on the one-third of sst_{2A}-expressing neurons that are not B5-I neurons. We therefore tested the effect of octreotide on *Bhlhb5*$^{-/-}$ mice, which lack B5-I neurons. Specifically, we reasoned that, if octreotide-induced scratching is due to inhibition of B5-I neurons, this treatment would have no effect in mice that lack these cells. As predicted, we observed very little scratching behavior upon intrathecal injection of 100 nM octreotide in *Bhlhb5*$^{-/-}$ mice (Figure 1F). This finding strongly suggests that octreotide-induced itch behavior is due to inhibition of B5-I neurons.

To further assess whether the octreotide-induced scratching was due to elevated itch (rather than a nociceptive response or a grooming behavior), we tested the effect of octreotide on pruritogen-evoked itch. For these experiments, we selected a very low dose of octreotide that had no significant effect on its own (3 ng) and tested its effect on chloroquine-induced itch. We found that intrathecal octreotide significantly increased the amount of time that mice spent biting at the injection site in response to intradermal chloroquine (Figure 1G). In contrast, this dose of intrathecal octreotide had no effect on acute nociceptive reflexes, as measured by hindpaw withdrawal latency

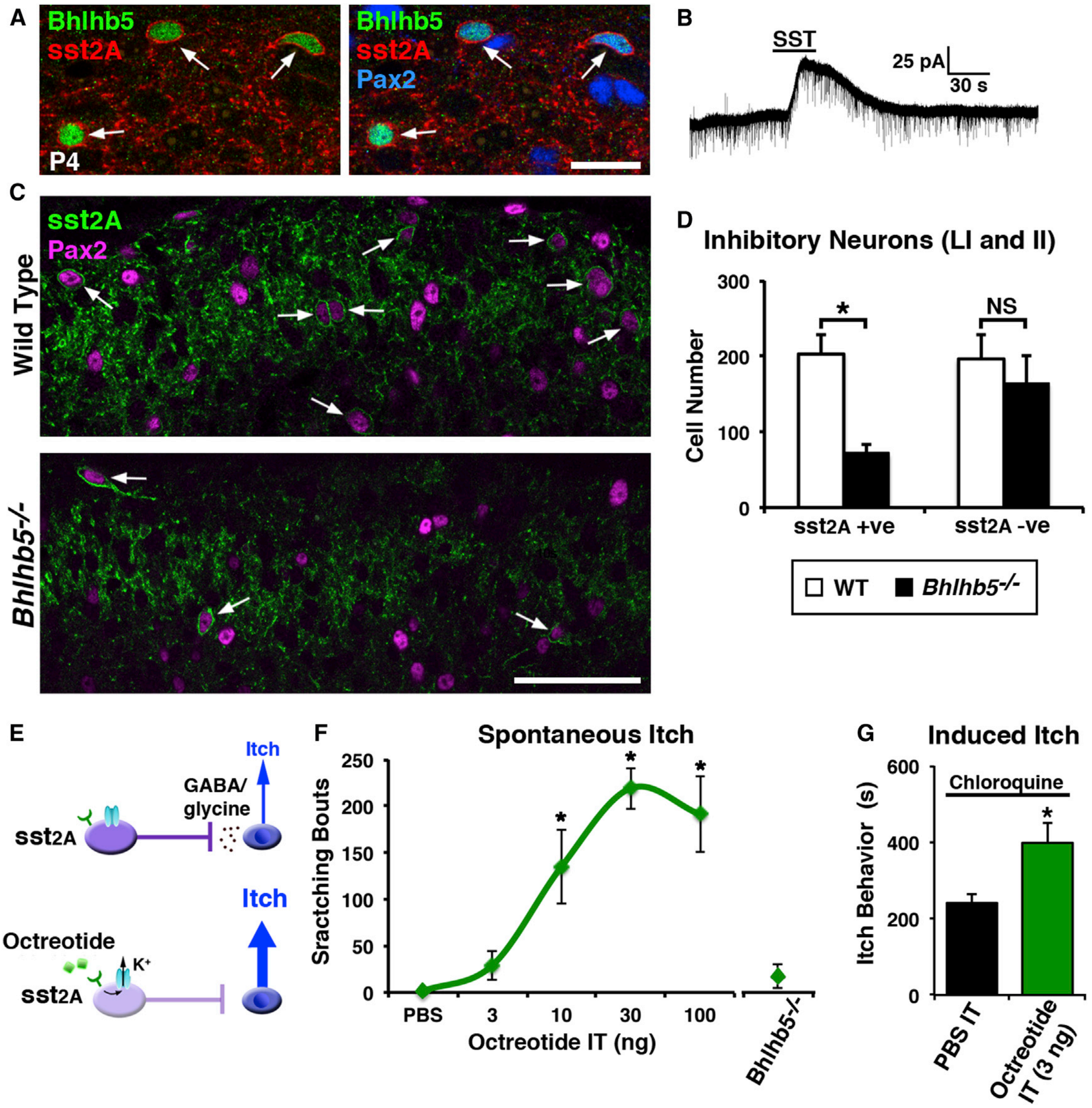

Figure 1. Acute Inhibition of B5-I Cells Results in Elevated Itch

(A) The inhibitory subset of Bhlhb5-expressing neurons in the superficial dorsal horn (B5-I neurons) coexpress sst_{2A}. Spinal cord sections from P4 mice were immunostained to reveal Bhlhb5 (green), sst_{2A} (red), and the inhibitory marker Pax2 (blue). The vast majority (~90%) of cells expressing Bhlhb5 and Pax2 in laminae I and II colabel with sst_{2A} (arrows). A single confocal optical section through laminae I-IIo is shown. Scale bar, 20 μm.

(B) Outward current was observed upon application of somatostatin (SST, 1 μM) to 50% of cells with the *Bhlhb5-cre* allele. (Note that the *Bhlhb5-cre* allele also labels other populations including excitatory neurons, which do not respond to SST.)

(C) The number of sst_{2A}-expressing neurons in dorsal horn is significantly diminished in *Bhlhb5*$^{-/-}$ mice. Spinal cord sections from 4- to 5-week-old wild-type (top) or *Bhlhb5*$^{-/-}$ (bottom) mice were immunostained to reveal sst_{2A} (green) and Pax2 (magenta). Approximately half of the inhibitory (Pax2-expressing) neurons in laminae I and II express sst_{2A} in wild-type mice (arrows), and these cells are dramatically reduced in *Bhlhb5* mutant mice. A single optical section is shown. Scale bar, 50 μm.

(D) Quantification of (C). There is a significant reduction (*$p < 0.05$) in the number of sst_{2A}-expressing neurons (sst_{2A} +ve) in laminae I-II of the dorsal horn in *Bhlhb5*$^{-/-}$ relative to wild-type (WT), with no significant change (NS) in the number of Pax2-positive inhibitory interneurons that do not express sst_{2A} (sst_{2A} –ve). Data are represented as mean + SD number of cells in laminae I-II per dorsal horn through 100 μm cord taken from L4 (n = 6 mice per genotype, 2 dorsal horns per mouse), analyzed by two-way ANOVA followed by pairwise comparison using the Holm-Sidak method.

(E) Schematic depicting inhibition of sst_{2A}-expressing interneurons with the somatostatin analog octreotide, resulting in elevated itch.

(F) Intrathecal administration of octreotide (3, 10, 30, or 100 ng in 5 μl vehicle) dose dependently evoked spontaneous scratching behavior in wild-type mice (n = 6–8 mice/treatment). *Bhlhb5*$^{-/-}$ mice receiving 100 ng of octreotide intrathecally showed very little scratching response. Intrathecal injections were confirmed by coinjection of 10% methylene blue (n = 8 mice). Total scratching bouts (mean ± SEM) were measured over a 30 min observation period. One-way ANOVA was used to compare mean scratch bouts between treatment groups followed by Tukey's post hoc test (* indicates significantly different than PBS, $p < 0.05$).

(G) Pruritogen-induced itch behavior was significantly enhanced following intrathecal octreotide (3 ng). Chloroquine (100 μg) was injected intradermally in the calf 30 min after treatment with either octreotide (i.t.) or vehicle (PBS; i.t.). Itch behavior was defined as the cumulative amount of time spent biting/licking the injection site over 30 min. Data are represented as mean ± SEM (*$p < 0.05$, Student's t test). Also see Figure S1 for the specificity of octreotide-mediated behavioral effects.

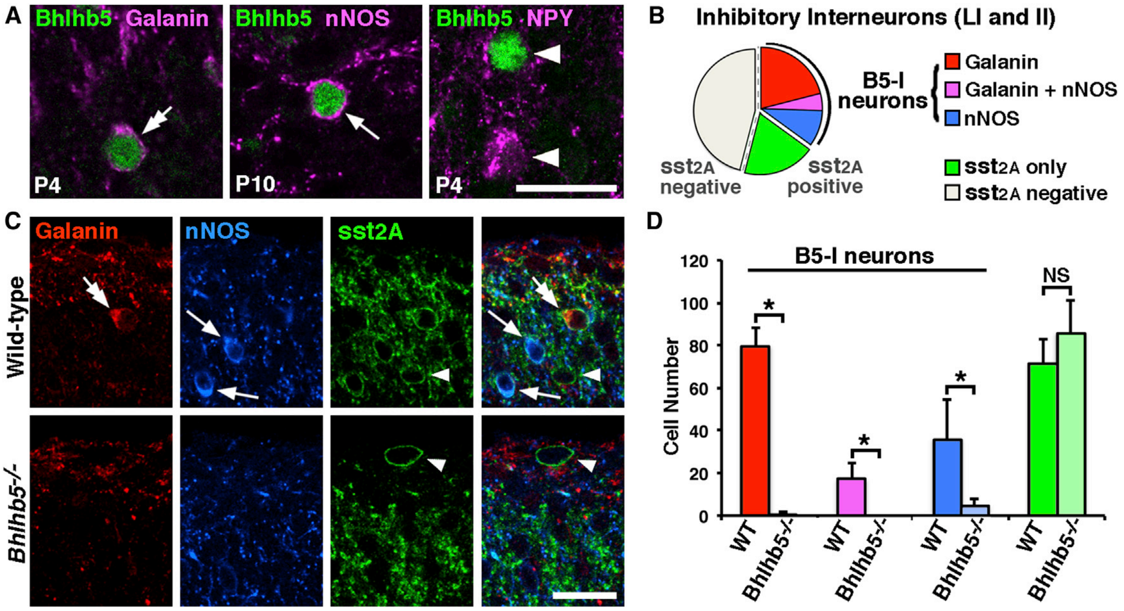

Figure 2. B5-I Neurons Belong to a Population that Expresses Galanin and/or nNOS

(A) The vast majority (95.4%, range 95.3%–95.7%, n = 3) of galanin-immunoreactive cells in laminae I-II (magenta) were Bhlhb5 immunoreactive (green; double arrow), and galanin-expressing cells accounted for 78.1% (range: 72.8%–86.4%) of B5-I neurons (image from lamina I-IIo). Numerous nNOS-immunoreactive cells (magenta) coexpress Bhlhb5 (green) as shown by the arrow (n = 3, image from lamina I). NPY seldom colocalized with Bhlhb5 (2.3% of NPY cells, range 1.1%–4.7%, n = 3; arrowheads illustrate cells that do not colocalize, lamina II). Images are single confocal optical sections from mice of indicated ages.

(B) Populations of inhibitory interneurons in laminae I-II of adult dorsal horn. Approximately 54% of these cells express sst_{2A} (sst_{2A} positive), and these can be further subdivided into classes based on expression of galanin (red), nNOS (blue), galanin and nNOS (magenta), or sst_{2A} only (neither galanin nor nNOS; green). B5-I neurons belong to the classes that express galanin and/or nNOS.

(C) Single optical sections of laminae I-II from wild-type or *Bhlhb5*$^{-/-}$ mice reveal a dramatic loss of the sst_{2A}-expressing cells that contain galanin (red, double-headed arrow) and nNOS (blue, arrows), while the sst_{2A}-expressing cells that contain neither galanin nor nNOS (sst_{2A} only) are still present (arrowheads). Scale bar, 20 μm.

(D) Quantification of (C) showing a significant reduction in cells expressing galanin (red), nNOS (blue), and galanin/nNOS (magenta). There was no significant change (NS) in the number of cells expressing sst_{2A} alone (green). Data are represented as mean + SD number of cells in laminae I-II per dorsal horn through 100 μm cord taken from L4 (n = 6 mice, genotype, 2 dorsal horns/mouse), analyzed by two-way ANOVA followed by pairwise comparison using the Holm-Sidak method (* indicates $p < 0.05$). See Figure S2 for large field views.

on a hot plate (Figure S1B). Furthermore, the effect of intrathecal octreotide was very likely mediated by spinal neurons (rather than the central terminals of primary afferents) since intradermal octreotide caused no itch-like behavior (Figure S1C). Together, these findings suggest that acute inhibition of B5-I neurons results in elevated itch.

Neurochemical Identification of B5-I Neurons

Sst_{2A}-expressing inhibitory neurons in laminae I-II can be further subdivided based on the presence or absence of galanin and neuronal nitric oxide synthase (nNOS), which are expressed in mostly nonoverlapping subsets (Figure 2B; Iwagaki et al., 2013; Tiong et al., 2011). To investigate whether B5-I neurons constitute one or more of these subsets, we performed immunostaining experiments. These experiments revealed that virtually all (~95%) of the galanin-expressing cells coexpress Bhlhb5 and that these account for 78% of the B5-I neurons (Figures 2A, left, and S2A). Likewise, many nNOS-expressing neurons coexpress Bhlhb5 (though the number is difficult to assess since nNOS is beginning to be expressed just as Bhlhb5 is being downregulated; Figures 2A, middle, and S2B). In contrast, Bhlhb5 was very seldom coexpressed with neuropeptide Y (NPY), a marker for a distinct inhibitory subpopulation (2% of NPY cells; Figures 2A, right, and S2C). These findings suggest that B5-I neurons correspond to two, mostly nonoverlapping subpopulations of inhibitory interneurons: those that express galanin and those that express nNOS.

We next investigated what happens to these populations in the *Bhlhb5*$^{-/-}$ mouse. Mice lacking *Bhlhb5* showed a dramatic loss of galanin- and nNOS-expressing populations, but there was no difference in the distribution of two other populations of inhibitory interneurons marked by NPY and parvalbumin, respectively (Figure S2D). To investigate this finding in more detail, we performed a quantitative analysis with the optical disector method (Polgár et al., 2004) on sections reacted for sst_{2A}, nNOS, galanin, and NeuN and stained with a nuclear marker (Figures 2B–2D and S2E). In wild-type mice, four subpopulations of sst_{2A}-expressing cells were identified: the first coexpressed galanin, a second coexpressed nNOS, a third (small) subpopulation coexpressed both galanin and nNOS, and a fourth expressed neither galanin nor nNOS (Figure 2B). In the *Bhlhb5*$^{-/-}$ mice, galanin-expressing cells were almost completely absent, while the number of sst_{2A}-expressing cells that contained nNOS but not galanin was substantially reduced (Figures 2C and 2D). In contrast, the number

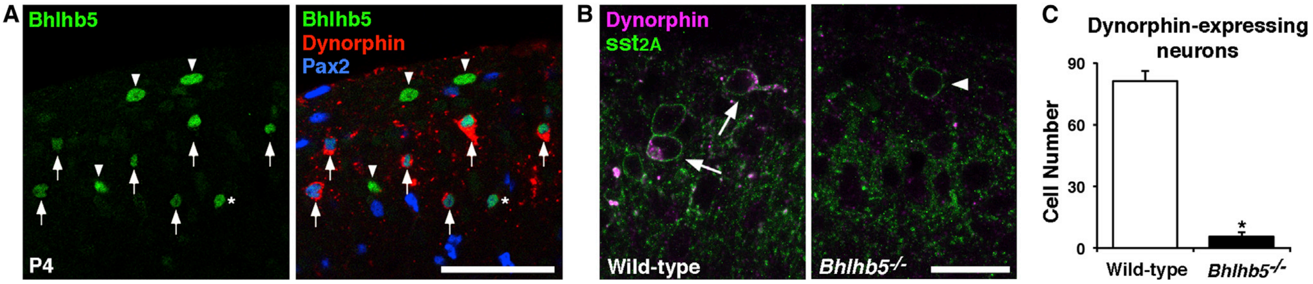

Figure 3. B5-I Neurons Express Dynorphin

(A) Spinal cord sections from P4 mice (n = 3) were immunostained to reveal Bhlhb5 (green), PPD (red), and Pax2 (blue). Virtually all (99%, range 98%–100%) cells expressing PPD and Pax2 were Bhlhb5 immunoreactive (arrows), and these accounted for the majority (85%, range 82.3%–87.95%) of B5-I cells at P4. Asterisk indicates a B5-I cell that does not express PPD. Pax2-negative Bhlhb5-expressing cells (presumed excitatory cells) are indicated with arrowheads. Scale bar, 20 μm. Similar results were observed using antibodies directed against Dynorphin B (Figure S3).

(B) Spinal cord sections from 4- to 5-week-old wild-type (left) or *Bhlhb5*$^{-/-}$ (right) mice immunostained for sst$_{2A}$ (green) and PPD (magenta). In wild-type mice, dynorphin is expressed in a subset of sst$_{2A}$-expressing neurons (arrows), whereas in *Bhlhb5*$^{-/-}$ mice, the sst$_{2A}$-expressing neurons that remain lack dynorphin (arrowheads). Images are single confocal optical sections.

(C) Quantification of (B) showing reduction in the mean number of dynorphin-expressing cells in laminae I and II in *Bhlhb5*$^{-/-}$ mice compared to wild-type controls. Data are represented as mean + SD number of cells per dorsal horn through 100 μm cord taken from L4 (n = 3 mice/genotype) and were analyzed by Student's t test (* indicates $p < 0.05$). Also see Figure S3.

of sst$_{2A}$-expressing cells that contained neither galanin nor nNOS did not differ significantly between wild-type and *Bhlhb5*$^{-/-}$ mice. This analysis of sst$_{2A}$-expressing interneurons, together with the previous quantification of inhibitory neurons that lack sst$_{2A}$ (Figure 1D), suggest that the galanin and nNOS populations of inhibitory neurons are severely depleted in *Bhlhb5*$^{-/-}$ mice, whereas all other inhibitory populations are unchanged.

B5-I Neurons Express the Kappa Opioid Dynorphin

The loss of galanin cells in the *Bhlhb5*$^{-/-}$ mice was of particular interest because these cells also express the kappa opioid dynorphin (Bröhl et al., 2008; Sardella et al., 2011), and there is precedent for the idea that kappa opioids inhibit itch (Inan and Cowan, 2004; Ko et al., 2003; Togashi et al., 2002). We therefore confirmed that dynorphin is expressed in B5-I neurons by reacting spinal cords from P4 mice with antibodies against Bhlhb5 and the dynorphin precursor, preprodynorphin (PPD; Figure 3A). As expected, we found that virtually all dynorphin-expressing neurons in laminae I-II were Bhlhb5 immunoreactive. We also assessed the number of dynorphin-expressing inhibitory interneurons in adult *Bhlhb5*$^{-/-}$ animals with antibodies against either PPD or dynorphin B, a cleavage product of the full-length dynorphin peptide. These experiments revealed an almost complete loss of dynorphin-expressing inhibitory interneurons in *Bhlhb5*$^{-/-}$ mice (Figures 3B, 3C, S3B, S3A and S3C), consistent with the finding that galanin-expressing neurons are largely absent in these mice.

Kappa Opioids Inhibit Itch in Response to a Variety of Pruritogens

The finding that *Bhlhb5*$^{-/-}$ mice lack spinal inhibitory neurons that release dynorphin raised the possibility that B5-I neurons normally inhibit itch in part through activation of the kappa opioid receptor (KOR). As a first step to test this idea, we investigated the effect of kappa agonists nalfurafine and U-50,488 (Figure 4A; Morgan and Christie, 2011; Wikström et al., 2005; Williams et al., 2013) on acute pruritogen-evoked itch behavior. To investigate whether kappa agonists inhibit itch mediated by MrgprA3/C11-expressing afferents, we quantified scratch bouts following intradermal injection of chloroquine into the nape of the neck of mice that had been pretreated with either nalfurafine (20 μg/kg) or vehicle. We found that nalfurafine significantly reduced chloroquine-induced itch behavior (Figures 4B and 4C), consistent with previous findings (Inan and Cowan, 2004). Likewise, nalfurafine significantly attenuated SLIGRL-mediated itch (Figure 4D). To extend these observations, we tested the effect of U-50,488 (3 mg/kg), a molecularly distinct kappa opioid agonist and observed a similar inhibition of chloroquine- and SLIGRL-induced scratching (Figures 4B, 4C, and 4D). Thus, kappa agonists inhibit itch mediated by MrgprA3/C11-expressing sensory neurons.

Recent studies have revealed that histamine-induced itch is different than chloroquine-induced itch in that it is mediated by a distinct subset of primary afferents (Roberson et al., 2013). We therefore tested the effect of KOR agonists on histamine-induced itch and found that scratching behavior was significantly reduced by nalfurafine, as previously reported (Togashi et al., 2002), as well as by U-50,488 (Figure 4E). Similarly, our experiments revealed that both nalfurafine and U-50,488 significantly reduced serotonin-induced itch (Figure 4F). Finally, we investigated a dry skin model of itch that develops following daily topical application of acetone/ether followed by water (AEW) (Akiyama et al., 2010b; Miyamoto et al., 2002). Both kappa agonists significantly reduced spontaneous scratching behavior produced by AEW treatment (Figure 4G). Importantly, neither U-50,488 (3 mg/kg) nor nalfurafine (20 μg/kg) had any significant effect on rotarod performance, indicating that their effects were not due to motor impairment (Figure S4A). Thus, KOR agonists significantly abate various types of pruritus, including histamine-dependent and histamine-independent itch.

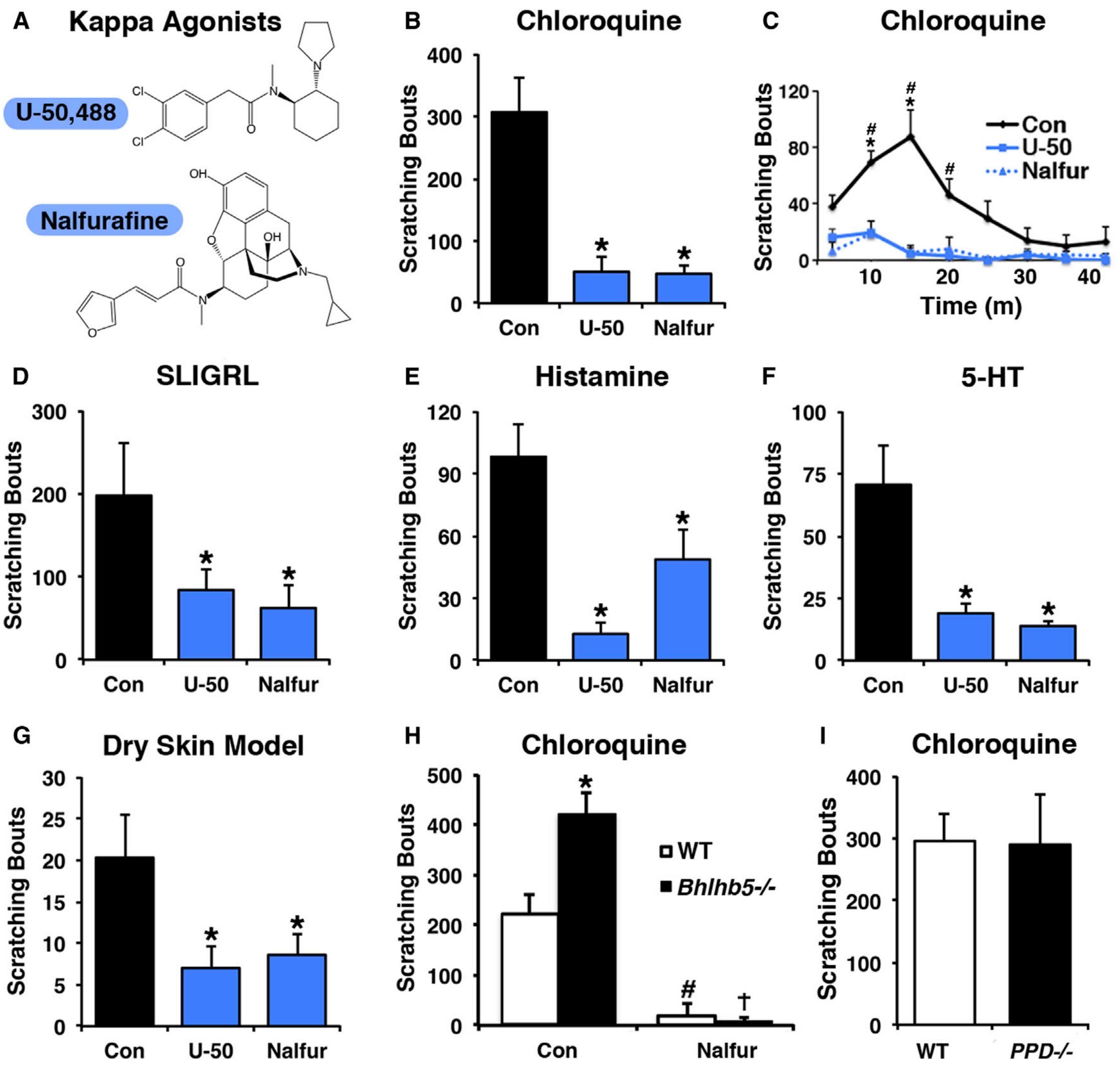

Figure 4. Systemic Administration of U-50,488 or Nalfurafine Inhibits Scratching Behavior
(A) Molecular structure of two KOR agonists, U-50,488 and nalfurafine (also called TRK-820).
(B–F) Pretreatment with U-50,488 (3 mg/kg) or nalfurafine (20 µg/kg) significantly attenuates scratching following intradermal injection of chloroquine (200 µg) (B and C), SLIGRL (100 nM) (D), Histamine (100 µg) (E), or 5-HT (30 µg) (F) relative to control (PBS). Data are represented as mean + SEM number of scratch bouts during the first 40 min after pruritogen injection (n = 8 mice/treatment). For (B), (D), (E), and (F), data were analyzed by one-way ANOVA and Tukey's post hoc test; *$p < 0.05$. For (C), data were analyzed by two-way ANOVA and Tukey's post hoc test; significant effects ($p < 0.05$) of U-50,488 (*) and nalfurafine (#) are indicated.
(G) Spontaneous scratching induced by AEW treatment was significantly reduced in mice pretreated with either U-50,488 or nalfurafine compared to controls receiving vehicle (PBS). Data are represented as mean + SEM number of scratch bouts recorded over a 1 hr observation period (n = 8 mice/treatment). Data were analyzed by one-way ANOVA and Tukey's post hoc test; *$p < 0.05$.
(H) Pretreatment with nalfurafine (20 µg/kg) significantly attenuated chloroquine-induced scratching behavior in both wild-type (WT; #$p < 0.05$) and *Bhlhb5*$^{-/-}$ (†$p < 0.05$) mice. * indicates significant difference ($p < 0.05$) in chloroquine-induced scratching between WT and *Bhlhb5*$^{-/-}$ mice. Experiments were performed using 4-week-old littermate pairs, prior to development of skin lesions (n = 8 mice/treatment). Data were analyzed by two-way ANOVA and Tukey's post hoc test.
(I) Itch sensitivity in constitutive *preprodynorphin*$^{-/-}$ (*PPD*$^{-/-}$) mice (Loacker et al., 2007) was analyzed by measuring the response to an intradermal injection of chloroquine into the nape of the neck. Scratching behavior did not significantly differ between treatment groups (n = 7 mice/genotype), suggesting that *PPD*$^{-/-}$ mice respond normally to acute pruritic stimuli. Data are represented as mean + SEM, and significance was assessed using a Student's t test.

These findings raised the possibility that decreased kappa opioid signaling, due to loss of dynorphin-expressing spinal interneurons, contributes to the abnormally elevated itch in *Bhlhb5*$^{-/-}$ mice. Thus, we reasoned that exogenous KOR agonists would relieve abnormal itch in these animals. As observed previously, we found that intradermal injection of chloroquine caused significantly more scratching in *Bhlhb5*$^{-/-}$ mice relative to littermate controls (Figure 4H; Ross et al., 2010). Importantly, pretreatment with nalfurafine almost completely abrogated scratching behavior in *Bhlhb5*$^{-/-}$ mice, consistent with the idea that abnormally elevated itch responses in these mice are partly due to decreased kappa tone in spinal cord (Figure 4H).

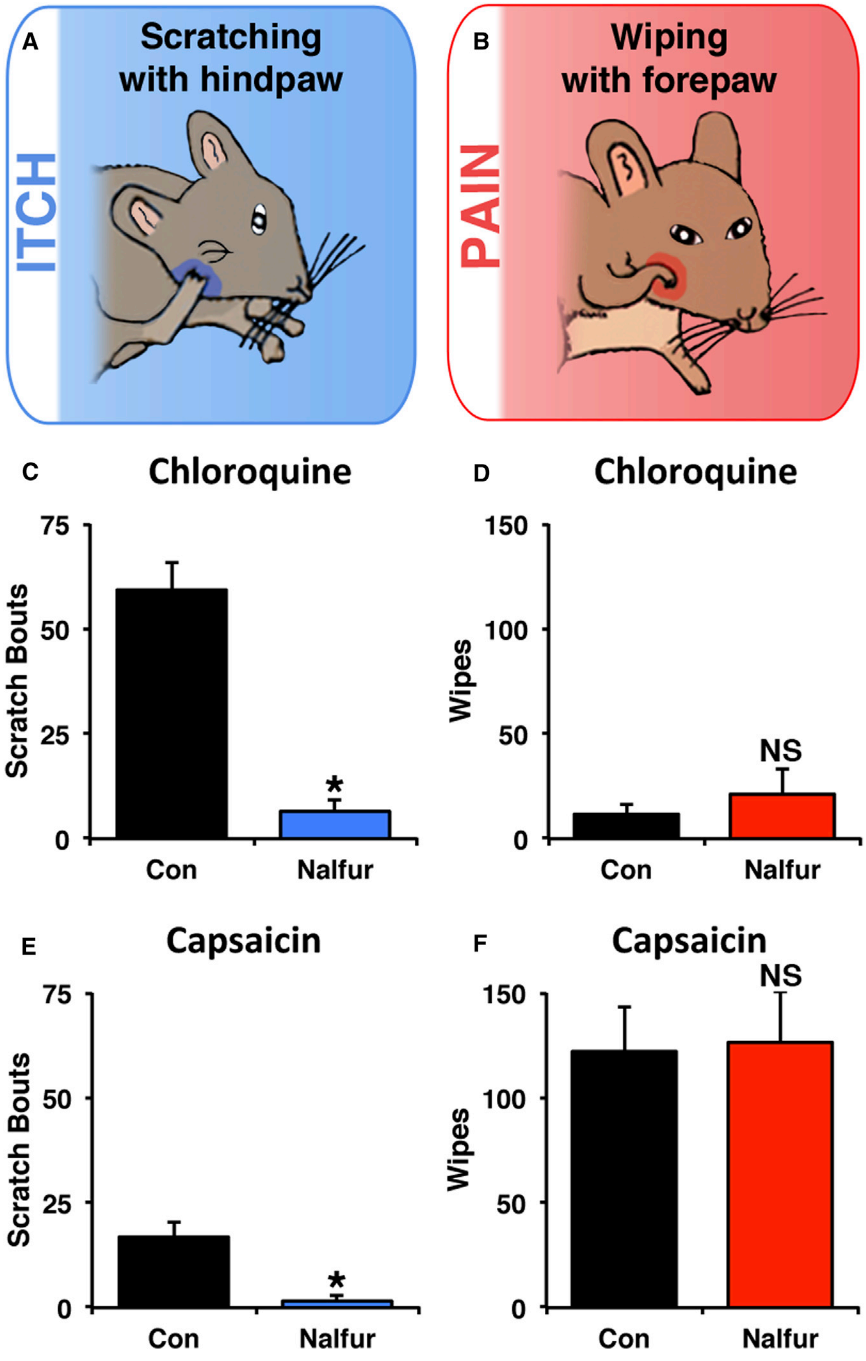

Figure 5. Kappa Agonists Selectively Inhibit Itch- but Not Pain-Associated Behaviors

(A and B) The cheek model was used to discriminate between pain- (B) and itch-related (A) behaviors. Wiping the cheek with the forepaw following an intradermal injection of a chemical stimulus is indicative of pain, while scratching the injection site with the hindlimb indicates itch.

(C) Intradermal chloroquine (100 μg) injected into the cheek elicited robust hindlimb-mediated scratching, and this was significantly attenuated in mice pretreated with nalfurafine (20 μg/kg) compared to control.

(D) Nalfurafine (20 μg/kg) had no significant effect on chloroquine-evoked wiping behavior.

(E) Intradermal injection of capsaicin (30 μg) into the cheek caused a small amount of hindlimb scratching, which was significantly reduced by nalfurafine (20 μg/kg).

(F) Nalfurafine (20 μg/kg) had no significant effect on capsaicin-induced wiping. For (C)–(F), data are shown as mean + SEM (n = 8 mice/treatment); $^*p < 0.05$, Student's t test; NS indicates no significant effect.

A question that remained unclear was whether the elevated itch in *Bhlhb5*$^{-/-}$ mice was simply due to the loss of dynorphin, or whether the absence of fast synaptic inhibition from B5-I neurons was also involved. To test whether constitutive loss of dynorphin was sufficient for abnormally elevated itch, we analyzed itch in mice lacking the dynorphin precursor PPD. We found that *PPD*$^{-/-}$ mice and their wild-type littermates showed no difference in pruritogen-induced itch behavior (Figure 4I). This observation suggests that the abnormally elevated itch observed in *Bhlhb5*$^{-/-}$ mice is not due to loss of spinal dynorphin alone, hinting at a key role for GABA and/or glycine in the inhibition of itch by B5-I neurons.

A consequence of the loss of B5-I neurons in *Bhlhb5*$^{-/-}$ mice is that these mice develop spontaneous skin lesions due to severe pathological itch. To test whether treatment with KOR agonists might provide therapeutic relief for neuropathic itch, we tested these drugs on *Bhlhb5*$^{-/-}$ mice with pruritic skin lesions. Systemic treatment with either U-50,488 or nalfurafine significantly reduced the amount of time *Bhlhb5*$^{-/-}$ mice spent biting and/or licking the site of lesion by 33% ± 14% and 40% ± 22%, respectively (Figures S4B and S4C), suggesting that kappa opioids have therapeutic potential for neuropathic itch conditions.

Kappa Opioids Are Selective for Itch

Because of the key role of mu opioids in inhibition of pain, numerous groups have assessed the potential role of KOR agonists as analgesics (Kivell and Prisinzano, 2010; Vanderah, 2010). While KOR agonists were found to be analgesic in some acute, inflammatory, and neuropathic pain tests, their analgesic efficacy at doses that do not affect motor coordination remains unclear (Leighton et al., 1988; Stevens and Yaksh, 1986). We therefore wondered whether a concentration sufficient to inhibit itch (i.e., 20 μg/kg of nalfurafine) is selective for pruritoception rather than nociception. To address this question, we used the cheek model (Figures 5A and 5B), in which pruritic agents elicit scratching with the hindlimb, whereas nociceptive substances

cause wiping with the forepaw (Shimada and LaMotte, 2008; Akiyama et al., 2010a). As expected, intradermal injection of chloroquine into the cheek induced robust hindlimb-mediated scratching with minimal wiping behavior, indicative of itch. Systemic pretreatment with nalfurafine led to an almost complete suppression of scratching, with no significant effect on wiping behavior (Figures 5C and 5D), in accordance with the idea that kappa agonists inhibit itch.

Next, to investigate the effect of kappa agonists on nociception, we injected capsaicin into the cheek. This treatment evoked intense site-directed wiping with little scratching, in keeping with the idea that pain is the predominant sensation elicited by capsaicin. Importantly, capsaicin-induced wiping was not affected by pretreatment with nalfurafine (Figure 5F), suggesting that nociceptive responses were unaffected by kappa opioid signaling. In contrast, the modest scratching in response to capsaicin was almost completely abolished following treatment with nalfurafine (Figure 5E). These results suggest that kappa opioid agonists, at least at low doses, can selectively inhibit itch with no effect on pain.

KOR Agonists and Antagonists Bidirectionally Modulate Itch at the Spinal Cord Level

The finding that systemic kappa opioids inhibit itch, together with our discovery that *Bhlhb5*$^{-/-}$ mice lack dynorphin-expressing spinal interneurons, raised the possibility that endogenous dynorphin and exogenous kappa opioids modulate itch through common neural circuits in the spinal cord. To test the idea that the inhibition of itch by kappa opioids is due, at least in part, to activation of spinal KORs, we manipulated KOR signaling in the spinal cord through intrathecal delivery of KOR agonists. Since intrathecal injections allowed us to target dermatomes L3–L5 (corresponding to the hindlimbs), itch behavior was assessed using the calf model (LaMotte et al., 2011), in which injection of a pruritic agent into the skin elicits a biting response (Figure 6A). Importantly, we found that intrathecal administration of either U-50,488 (10 μg) or nalfurafine (40 ng) to the lumbar spinal cord significantly reduced chloroquine-evoked biting (Figure 6B). These findings suggest that activation of KORs in the spinal cord is sufficient to inhibit itch.

A key question is the identity of the cellular targets for kappa opioids within the spinal cord. Though the central processing of itch is not clearly understood, recent work has suggested that itch information is sequentially relayed by at least two types of spinal interneurons (Npra-expressing neurons followed by GRPR-expressing neurons) before being transmitted to the brain (Mishra and Hoon, 2013). We therefore investigated whether kappa opioids act upstream or downstream of GRPR-expressing neurons by testing the effect of nalfurafine on GRP-mediated itch. Intrathecal injection of GRP caused robust scratching that was significantly reduced by nalfurafine (Figure 6C). This finding suggests that kappa agonists mediate their effect (either directly or indirectly) on GRPR-expressing neurons, or on neurons downstream of GRPR activation in the spinal cord.

Next, we reasoned that if B5-I neurons normally release dynorphin to inhibit itch, then blocking endogenous KOR signaling in the dorsal horn might result in elevated itch. To test this idea, we investigated whether treatment with the KOR antagonists norbinaltorphimine (norBNI) or 5′-guanidinonaltrindole (5′GNTI; Figure 6D) could trigger an enhanced response to chloroquine in the calf. We found that chloroquine-induced biting was significantly increased by intrathecal norBNI. Likewise treatment with 5′GNTI intrathecally increased the amount of chloroquine-induced biting relative to control (Figure 6E). The finding that blocking KOR signaling increases itch response to chloroquine suggests that endogenous spinal dynorphin normally functions to dampen itch. Together, these results show that modulating opioid tone in the spinal cord can bidirectionally alter itch sensitivity—increasing kappa opioid signaling causes decreased itch, whereas decreasing kappa opioid signaling results in increased itch.

B5-I Neurons Mediate Inhibition of Itch by Chemical Counterstimuli

In light of the finding that B5-I neurons function to inhibit itch, we wished to characterize these cells in more detail. We performed patch-clamp recordings from lamina II neurons genetically labeled with the *Bhlhb5-cre* allele (Figure 7A). Since this allele labels a somewhat broader population than those that we define as B5-I neurons, we used hyperpolarization in response to somatostatin to confirm that we were recording from B5-I neurons. Four basic firing patterns can be identified in lamina II interneurons in response to injection of depolarizing current: tonic, delayed, phasic/transient, and single spiking (Graham et al., 2007; Heinke et al., 2004; Ruscheweyh and Sandkühler, 2002). We found that the majority (29 out of 34) of B5-I neurons showed tonic firing (Figures 7B and S5A) and may therefore function as integrators. Neurons can be classified based on morphology and previous studies have described several types including vertical, islet, central, and radial, although many cells cannot be classified according to this scheme (Grudt and Perl, 2002; Yasaka et al., 2007, 2010). To determine whether B5-I neurons belonged to any of these subsets, we reconstructed B5-I neurons. Though B5-I neurons did not fit strictly into a single class, the majority were either central or unclassified, with axons and dendrites mainly restricted to lamina II (Figure 7C). Thus, B5-I neurons are likely to be involved in integrating sensory input within the substantia gelatinosa.

One of the hallmarks of itch is that it is relieved by a variety of counterstimuli, such as scratching, noxious chemicals, or menthol (Bromm et al., 1995; Ward et al., 1996; Yosipovitch et al., 2007). While the neural basis for this phenomenon is unknown, it has been suggested that counterstimuli reduce itch through activation of spinal inhibitory interneurons (Akiyama et al., 2011; Ma, 2010; Patel and Dong, 2010; Ross, 2011; Bautista et al., 2014). Based on our findings, B5-I neurons seemed well positioned to mediate the inhibition of itch by counterstimuli. If so, we reasoned that they would receive input (either directly or indirectly) from primary afferents that mediate the counterstimuli.

Capsaicin, mustard oil, and menthol activate discrete subsets of primary afferents (those that express TrpV1, TrpA1, and TrpM8, respectively). Since topical treatment with any of these substances can inhibit itch, we tested whether B5-I neurons receive input from primary afferents that express TrpV1, TrpA1, or TrpM8 (Figure 7D). Upon application of capsaicin to depolarize TrpV1-expressing afferents, we saw a significant increase

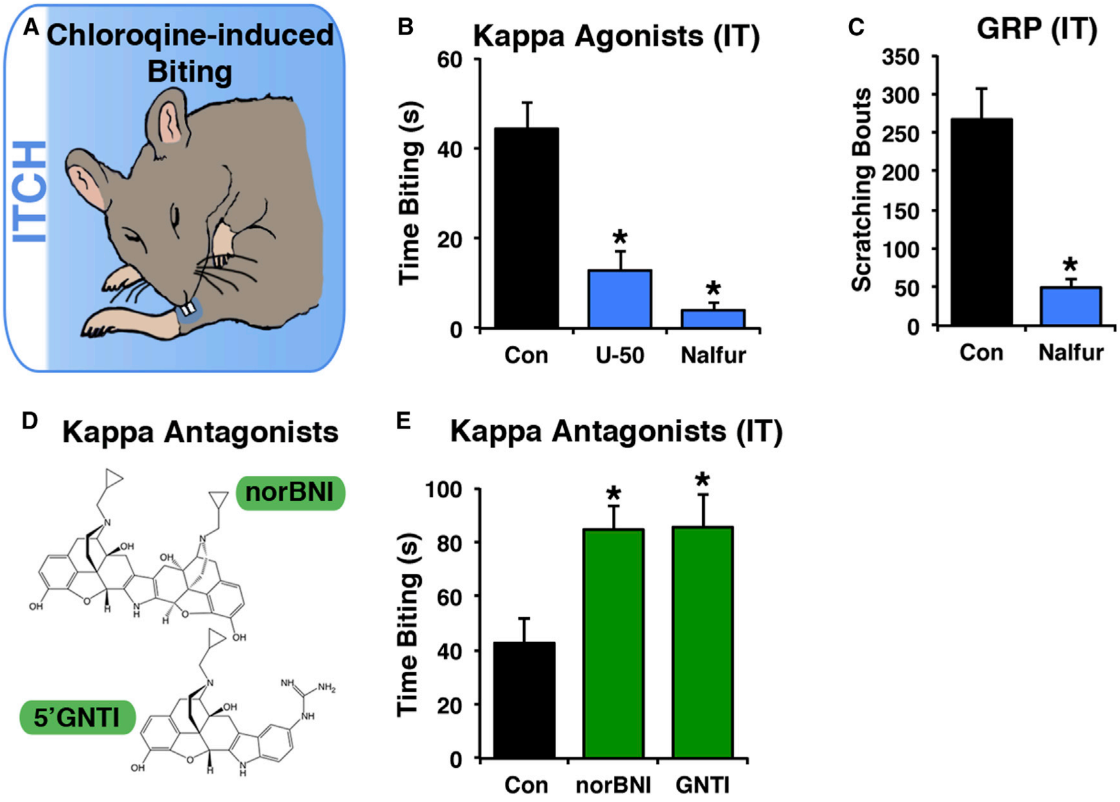

Figure 6. Modulation of Kappa Opioid Tone in the Spinal Cord Bidirectionally Modulates Itch Behavior

(A) The calf model was used to evaluate itch-related behaviors. In this model, a pruritogen is injected into the skin of the calf and biting is indicative of itch.

(B) Intrathecal administration of 10 μg U-50,488 or 40 ng nalfurafine (doses 60-fold lower than those used above for systemic administration) significantly reduced chloroquine-induced biting. Data are presented as mean + SEM (n = 6–8 mice/treatment). * indicates significant difference ($p < 0.05$) between treatment and control using one-way ANOVA and Tukey's post hoc test.

(C) Nalfurafine significantly reduces GRP-induced itch behavior. Intrathecal GRP-evoked scratching behavior was significantly attenuated when GRP (0.1 nmol) was coinjected with nalfurafine (40 ng) compared to PBS. Data are represented as mean + SEM number of scratch bouts recorded over a 30 min observation period (n = 7 mice/treatment). Data were analyzed by a Student's t test; * indicates $p < 0.05$.

(D) Molecular structure of two kappa opioid receptor antagonists, norbinaltorphimine (norBNI) and 5′-guanidinonaltrindole (5′GNTI).

(E) Chloroquine-induced biting was significantly elevated after intrathecal administration of the kappa opioid antagonists 5′GNTI (1 μg) or nor-BNI (1 μg). Data are presented as mean + SEM (n = 6–8 mice/treatment). * indicates significant difference ($p < 0.05$) between treatment and control using a one-way ANOVA and Tukey's post hoc test.

in the frequency of spontaneous excitatory postsynaptic currents (sEPSCs) in 80% (4 of 5) of B5-I neurons, with an average 7.8-fold increase in EPSC frequency (Figures 7E and 7F). Moreover, a significant increase in mEPSC frequency was likewise observed in the presence of tetrodotoxin (TTX) to block action potential propagation, suggesting that B5-I neurons receive direct input from capsaicin-sensitive sensory neurons (Figure S5B). Similarly, allyl isothiocyanate, a key component of mustard oil, resulted in increased sEPSC frequency in 86% (6 out of 7) B5-I neurons, with an average increase of 3.3-fold (Figures 7G and 7H). Finally we observed that sEPSC frequency was also significantly increased 2.5-fold by menthol in 90% (9 out of 10) of B5-I neurons (Figures 7I and 7J). Once again, the increase in mEPSC frequency in response to capsaicin, mustard oil, and menthol was also observed in the presence of tetrodotoxin (TTX) to block action potentials, suggesting that TrpA1- and TrpM8-expressing afferents directly innervate B5-I neurons (Figures S5B, S5C, and S5D).

The finding that B5-I neurons receive direct input from sensory neurons that respond to capsaicin, mustard oil, and menthol is consistent with the idea that B5-I neurons mediate the inhibition of itch by chemical counterstimuli. To directly test this possibility, we developed a mouse model of inhibition of itch by menthol. When wild-type mice were treated with 8% menthol (topically) on the cheek, this caused a significant reduction in subsequent chloroquine-induced scratching. In contrast, *Bhlhb5*$^{-/-}$ mice showed no significant inhibition of itch by menthol (Figure 8A). These findings suggest that B5-I neurons are required for the inhibition of itch by menthol (Figure 8B).

DISCUSSION

While our everyday experience that itch is relieved by counterstimulation indicates that itch is under inhibitory control, the neural basis for this phenomenon has remained obscure and neuromodulators of itch have not been identified. Here we begin to shed

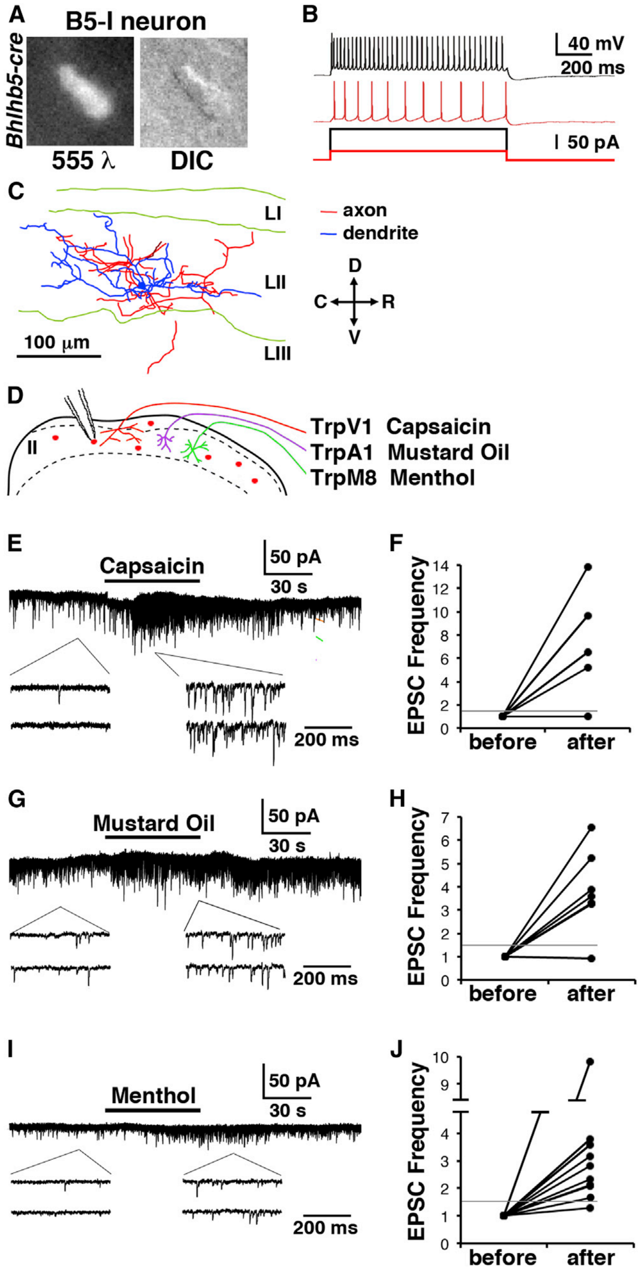

Figure 7. B5-I Neurons Are Innervated by Sensory Neurons that Respond to Capsaicin, Mustard Oil, and Menthol

(A) *Bhlhb5-cre* neurons were recorded in patch-clamp experiments; fluorescence (left) and IR-DIC (right) image. B5-I neurons had input resistances of 641.7 ± 71.7 MΩ and a resting membrane potential of −63.3 ± 1.4 mV. Every neuron exhibited EPSCs at an average amplitude of 7.9 ± 0.6 pA at a holding potential of −70 mV. No IPSCs were observed because the reversal potential for IPSCs was near −70 mV.

(B) Representative firing pattern of B5-I neuron in response to depolarizing current pulse of 25 (red) and 100 (black) pA.

(C) Morphology of B5-I neurons. Neurolucida reconstructions revealed that most B5-I neurons are central (illustrated) or unclassified. Blue, dendrites; red, axons.

(D) Schematic illustrating experimental setup to test whether B5-I neurons receive input from TrpV1-, TrpA1-, or TrpM8-expressing neurons.

(E–J) Presynaptic effect of 2 µM capsaicin (E), 100 µM allyl isothiocyanate (mustard oil) (G), and 500 µM menthol (I). The majority of B5-I neurons showed a significant increase in sEPSC frequency after bath application of capsaicin (80%; F), mustard oil (86%; H), and menthol (90%; J). See Figure S5 for further electrophysiological analysis of B5-I neurons.

light on this issue by identifying a neuronal subtype in the spinal cord—B5-I neurons—that inhibits itch. We discover that B5-I neurons correspond to specific neurochemical populations and show that they are the major source of the endogenous kappa opioid dynorphin in the dorsal horn. Our data suggest that kappa opioids selectively inhibit itch without affecting pain. Indeed, modulation of kappa opioid tone in the spinal cord can bidirectionally control itch sensitivity, implying that dynorphin acts as a neuromodulator. Finally, we demonstrate that B5-I neurons are innervated by capsaicin-, mustard oil-, and menthol-responsive primary afferents and are required for inhibition of itch by menthol. These data suggest that B5-I neurons mediate the inhibition of itch by chemical counterstimuli (Figure 8B).

Dorsal Horn Interneurons

Inhibitory interneurons, which use GABA and/or glycine, account for 25%–30% of neurons in laminae I-II (Polgár et al., 2003, 2013b) and are thought to perform several distinct roles in sensory processing (Hughes et al., 2012; Ross, 2011; Sandkühler, 2009). To understand how these cells modulate somatosensory input, it is essential to distinguish different functional populations among them (Graham et al., 2007; Todd, 2010). The most widely accepted scheme for classifying superficial dorsal horn interneurons was developed by Grudt and Perl (2002), who identified four main groups, based largely on morphological criteria. However, though others have used this scheme, ~30% of neurons in these studies could not be classified based on morphology (Heinke et al., 2004; Maxwell et al., 2007; Yasaka et al., 2007, 2010). Moreover, with the exception of islet cells, inhibitory neurons are morphologically diverse (Yasaka et al., 2010). Thus, morphology does not appear to be particularly useful for defining inhibitory interneuron subpopulations.

An alternative approach uses the wide array of neuropeptides, receptors, and other proteins that are differentially expressed by dorsal horn neurons (Polgár et al., 2013a, 2013b). We previously found that ~50% of inhibitory cells in laminae I-II express sst_{2A}, and these can be further subdivided into subpopulations that contain galanin and/or nNOS (which constitute ~60% of the sst_{2A}-expressing cells and therefore approximately one-third of all the inhibitory neurons). The galanin cells coexpress PPD and are the major source of dynorphin in the superficial laminae (Bröhl et al., 2008; Sardella et al., 2011). In addition, we identified two other nonoverlapping groups among inhibitory interneurons that lack sst_{2A} (NPY- and parvalbumin-expressing cells). There is already evidence that these neurochemical classes differ, both in their responses to noxious stimuli and in their postsynaptic targets (Todd, 2010; Hughes et al., 2012; Polgár et al., 2013b). The present results provide further evidence in support of this classification scheme, since *Bhlhb5*$^{-/-}$ mice show a loss of

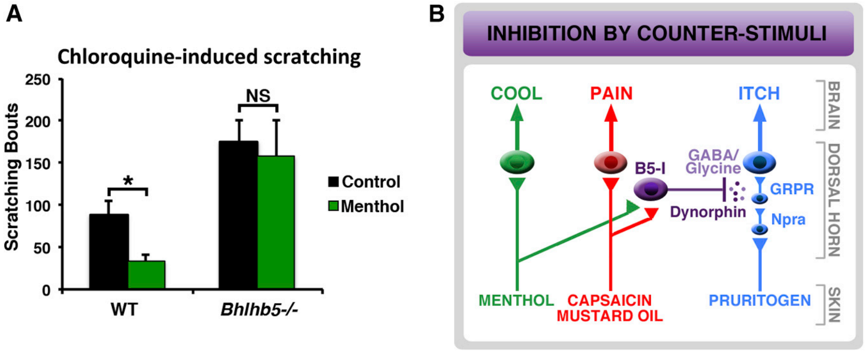

Figure 8. B5-I Neurons Mediate Inhibition of Itch by Chemical Counterstimuli

(A) Mice were treated topically on the cheek with 10 μl of either 8% menthol or PBS (control). Menthol treatment resulted in modest wiping behavior that ceased within 5 min. Ten minutes after initial treatment, chloroquine (100 μg) was injected intradermally into the cheek and hindlimb-mediated scratching was quantified. Menthol significantly reduced subsequent chloroquine-induced scratching behavior in wild-type mice, but not in *Bhlhb5*$^{-/-}$ mice. Data are presented as mean + SEM (n = 8 mice/treatment). * indicates significant difference ($p < 0.05$) between treatment and control using two-way ANOVA and Tukey's post hoc test. Note that there was also a significant difference in chloroquine-induced scratching as a function of genotype, as seen previously (Ross et al., 2010).

(B) Model: menthol-, capsaicin-, and mustard oil-sensitive sensory neurons inhibit itch via B5-I neurons in dorsal horn. B5-I neurons release GABA and/or glycine, which may cause acute inhibition of itch, as well as dynorphin, which acts as an inhibitory neuromodulator. Though the postsynaptic targets of B5-I neurons are not known, the finding that kappa agonists inhibit GRP-mediated itch implies that kappa opioids act on or downstream of GRPR neurons.

inhibitory interneurons that is apparently restricted to neurochemically defined populations.

Identification and Characterization of B5-I Neurons

We find that B5-I neurons correspond to two (mostly nonoverlapping) subpopulations—those that coexpress galanin and dynorphin and those that express nNOS. The subpopulation of B5-I neurons that expresses galanin/dynorphin likely uses GABA as its fast transmitter (Simmons et al., 1995), whereas the B5-I neurons that express nNOS are thought to release GABA and glycine (Spike et al., 1993). Since relief of itch by counterstimuli begins almost instantaneously, we favor the idea that this component is mediated by fast-acting inhibitory transmitters. In contrast, dynorphin, which modulates neuronal activity via G protein-coupled receptors, may underlie prolonged suppression of itch.

A key finding from our study is that the loss of B5-I neurons (which results in an almost complete absence of dynorphin in the spinal cord) has a different phenotypic outcome than loss of dynorphin alone. Thus, *Bhlhb5*$^{-/-}$ mice show dramatically elevated itch, whereas *PPD*$^{-/-}$ mice display normal itch sensitivity. This distinction implies that an organism can compensate for the loss of dynorphin, but not for the loss of dynorphin-expressing neurons in the dorsal horn. We speculate that neuromodulatory mechanisms may be particularly amenable to homeostatic compensation (Doi and Ramirez, 2010). In keeping with this idea, mice lacking either enkephalin or the mu opioid receptor have subtle pain phenotypes (König et al., 1996; Matthes et al., 1996), despite the fact that mu opioids are among the most effective analgesics. Adaptation also occurs in response to chronic opioid overexposure, as shown by the tolerance observed in humans and animal models following long-term treatment with opioid analgesics (Morgan and Christie, 2011; Williams et al., 2013). These examples underscore the idea that the nervous system is robust in its ability to adjust neural circuit function over time when opioid neuromodulatory function is abnormal. In contrast, neural circuits in the dorsal horn are unable to normalize itch sensitivity when B5-I neurons are lacking, emphasizing the fundamental requirement of this neuronal subtype for the normal manifestation of itch.

B5-I Cells Mediate the Inhibition of Itch by Counterstimuli

The idea that spinal interneurons are involved in sharpening acuity between sensory modalities has been proposed by us and others (Ma, 2010; Ross, 2011; Zheng et al., 2010; Prescott et al., 2014). In keeping with this idea, conditional loss of VGLUT2 in subsets of primary afferents resulted in mice showing decreased nociceptive responses but heightened pruritic responses, suggesting a role for inhibitory neurons in the suppression of itch by noxious input (Lagerström et al., 2010; Liu et al., 2010). However, the specific identity of spinal interneurons that mediate this type of inhibition was unknown. The B5-I neurons that we describe here are well suited for this role since they receive direct input from primary afferents that are known to suppress itch. In addition, we now provide direct evidence that B5-I neurons suppress itch, since acute inhibition of these cells results in spontaneous scratching. Finally, we show that, whereas menthol inhibits itch in wild-type mice, it does not do so in mice that lack B5-I neurons. Together, these data suggest that B5-I neurons mediate the inhibition of itch by menthol and likely other chemical counterstimuli.

Our findings also suggest that specific neuromodulators may be involved in selectively tuning different types of somatosensory input. This has strong precedent elsewhere in the nervous system, where kappa and mu and opioids have distinct (and often opposing) neuromodulatory roles. In the limbic system, mu opioids are euphoric while kappa opioids are dysphoric (Pfeiffer et al., 1986; Schlaepfer et al., 1998). In the hypothalamus, mu and kappa opioids have opposing effects on body temperature (Xin et al., 1997). Indeed, mu and kappa receptor-expressing neurons have been found to inhibit one another directly, thereby mediating the mutually antagonistic effects in modulation of pain by the nucleus raphe magnus (Pan et al., 1997). Now parallels are beginning to emerge in the spinal cord, where mu agonists specifically target nociception, and kappa agonists, as we show here, selectively inhibit itch.

Kappa Opioid Agonists as a Treatment for Pruritus

Pruritus is one of the most common adverse effects following spinal administration of mu opioid agonists, affecting >50% of patients receiving epidural morphine (Kjellberg and Tramèr,

2001). Naltrexone, a mu opioid receptor antagonist, is commonly coadministered to reduce the intensity of pruritus, but its use is limited due to its antianalgesic effects (Abboud et al., 1990). Interestingly, nalbuphine, a mixed kappa opioid agonist/mu opioid antagonist, is extremely effective in reducing postoperative pruritus (Liao et al., 2011). Unlike naltrexone, nalbuphine does not significantly antagonize the analgesic effects of epidural morphine. This suggests that the ability of nalbuphine to reduce morphine-induced itch is largely due to its KOR agonist activity. Consistent with this, KOR agonists have been shown to reduce morphine-induced itch in monkey (Ko et al., 2003). These findings raise the possibility that coadministration of mu and kappa opioids (or the use of agonists with affinities for both receptors) may offer pain relief without causing itch.

An important question raised by our study is the identity of the dorsal horn neurons that respond to kappa opioids. KORs have been detected on some neurons in laminae I-II (Arvidsson et al., 1995), and approximately 15% of lamina II neurons are hyperpolarized by kappa opioids (Eckert and Light, 2002; Peckys and Landwehrmeyer, 1999). While we do not yet know the identity of these cells, our finding that GRP-evoked itch is attenuated by nalfurafine is consistent with the idea that kappa opioids directly inhibit GRPR-expressing spinal interneurons. Alternatively, kappa opioids may act downstream, targeting as-yet-unidentified interneurons or projection neurons that mediate itch. Identifying the dorsal horn neuronal subtype(s) that express the KOR will be of great interest, as these cells may represent a point of convergence between neural circuits mediating itch and those responsible for inhibition of itch by counterstimuli.

Several clinical trials have shown that nalfurafine is effective in reducing itch in patients with chronic renal failure (Kumagai et al., 2012; Wikström et al., 2005). Furthermore, nalfurafine is well tolerated, and dysphoria is not reported even after 1 year of treatment. Our study provides insight into the mechanism through which kappa agonists inhibit itch, raising the possibility that this class of drugs may be broadly applicable as antipruritics. Thus, kappa agonists may have therapeutic potential for the treatment of pruritus resulting from a wide range of dermatological and systemic diseases.

EXPERIMENTAL PROCEDURES

Animals and Behavioral Experiments

Most behavioral tests were carried out on 6- to 8-week-old male C57bl/6 mice. Experiments that involved *Bhlhb5*$^{-/-}$ mice (Ross et al., 2010) were performed on 4- to 5-week-old mice that did not have skin lesions, unless otherwise stated. To generate age-matched wild-type and *Bhlhb5*$^{-/-}$ mice, we harem mated *Bhlhb5*$^{-/+}$ mice and wild-type and *Bhlhb5*$^{-/-}$ offspring from the resulting litters were used. In all experiments, the observer was blind to genotype and/or treatment. The use of animals was approved by the Institutional Animal Care and Use Committee of the University of Pittsburgh and/or the Ethical Review Process Applications Panel of the University of Glasgow. Experiments performed in A.J.T.'s lab were in accordance with the UK Animals (Scientific Procedures) Act 1986. Further details are provided in the Supplemental Experimental Procedures.

Immunocytochemistry

Immunocytochemistry was performed using standard protocols. The antibodies used in the study are listed in Table S1, and details of image analysis are given in the Supplemental Experimental Procedures.

Electrophysiology

Laminectomies were performed on 4- to 6-week-old mice was performed, and the spinal cord was excised to prepare parasagittal or transverse slices. We defined neurons as being sensitive to a particular drug when the synaptic response was altered by more than ±50%. Biocytin-filled cells were reconstructed with Neurolucida (MicroBrightField). Further details are provided in the Supplemental Experimental Procedures.

SUPPLEMENTAL INFORMATION

Supplemental Information includes Supplemental Experimental Procedures, five figures, and one table and can be found with this article online at http://dx.doi.org/10.1016/j.neuron.2014.02.046.

AUTHOR CONTRIBUTIONS

A.P.K., X.C., C.R.F., G.M.H., C.S.B., and E.S.S. performed and analyzed behavioral experiments with supervision from S.E.R. E.P., D.C., and S.S. performed and analyzed immunohistochemical experiments with supervision from A.J.T. J.H., L.M.S., and S.K. performed and analyzed electrophysiological experiments with supervision from H.R.K. and S.E.R. H.N., C.S., M.W., T.F., and T.K. contributed reagents. A.P.K., E.P., J.H., L.M.S., A.J.T., and S.E.R. wrote the paper.

ACKNOWLEDGMENTS

This research was supported by NIH grants R01 AR063772 and R21 AR064445 to S.E.R. and by grants to A.J.T. from the MRC (MR/L003430/1) and BBSRC (BB/J001082). Part of this work was supported by a grant from the Rita Allen Foundation to S.E.R., who is a Rita Allen Foundation Pain Scholar. Additional funding came from the Virginia Kaufman Endowment Fund No. 1 of the University of Pittsburgh to S.E.R., the Competitive Medical Research Fund of Pittsburgh to S.E.R., a Whitehall Foundation Research Grant to S.E.R., and the Wellcome Trust to A.J.T. We thank S. Fulton, L. De Souza, E. Burrage, and R. Kerr for expert technical assistance, Dr. Z. Puskár for helpful advice, and Dr. P. Ciofi for the gift of dynorphinB antibody. H.N. is a partial patent owner of nalfurafine (WO 93/15081); all other authors declare no conflict of interest.

Accepted: February 25, 2014
Published: April 10, 2014

REFERENCES

Abboud, T.K., Lee, K., Zhu, J., Reyes, A., Afrasiabi, A., Mantilla, M., Steffens, Z., and Chai, M. (1990). Prophylactic oral naltrexone with intrathecal morphine for cesarean section: effects on adverse reactions and analgesia. Anesth. Analg. *71*, 367–370.

Akiyama, T., Carstens, M.I., and Carstens, E. (2010a). Differential itch- and pain-related behavioral responses and μ-opoid modulation in mice. Acta Derm. Venereol. *90*, 575–581.

Akiyama, T., Carstens, M.I., and Carstens, E. (2010b). Spontaneous itch in the absence of hyperalgesia in a mouse hindpaw dry skin model. Neurosci. Lett. *484*, 62–65.

Akiyama, T., Iodi Carstens, M., and Carstens, E. (2011). Transmitters and pathways mediating inhibition of spinal itch-signaling neurons by scratching and other counterstimuli. PLoS ONE *6*, e22665.

Arvidsson, U., Riedl, M., Chakrabarti, S., Vulchanova, L., Lee, J.H., Nakano, A.H., Lin, X., Loh, H.H., Law, P.Y., Wessendorf, M.W., et al. (1995). The kappa-opioid receptor is primarily postsynaptic: combined immunohistochemical localization of the receptor and endogenous opioids. Proc. Natl. Acad. Sci. USA *92*, 5062–5066.

Bautista, D.M., Wilson, S.R., and Hoon, M.A. (2014). Why we scratch an itch: the molecules, cells and circuits of itch. Nat. Neurosci. *17*, 175–182.

Bröhl, D., Strehle, M., Wende, H., Hori, K., Bormuth, I., Nave, K.A., Müller, T., and Birchmeier, C. (2008). A transcriptional network coordinately determines

transmitter and peptidergic fate in the dorsal spinal cord. Dev. Biol. *322*, 381–393.

Bromm, B., Scharein, E., Darsow, U., and Ring, J. (1995). Effects of menthol and cold on histamine-induced itch and skin reactions in man. Neurosci. Lett. *187*, 157–160.

Doi, A., and Ramirez, J.M. (2010). State-dependent interactions between excitatory neuromodulators in the neuronal control of breathing. J. Neurosci. *30*, 8251–8262.

Eckert, W.A., 3rd, and Light, A.R. (2002). Hyperpolarization of substantia gelatinosa neurons evoked by mu-, kappa-, delta 1-, and delta 2-selective opioids. J. Pain *3*, 115–125.

Graham, B.A., Brichta, A.M., and Callister, R.J. (2007). Moving from an averaged to specific view of spinal cord pain processing circuits. J. Neurophysiol. *98*, 1057–1063.

Grudt, T.J., and Perl, E.R. (2002). Correlations between neuronal morphology and electrophysiological features in the rodent superficial dorsal horn. J. Physiol. *540*, 189–207.

Han, L., Ma, C., Liu, Q., Weng, H.J., Cui, Y., Tang, Z., Kim, Y., Nie, H., Qu, L., Patel, K.N., et al. (2013). A subpopulation of nociceptors specifically linked to itch. Nat. Neurosci. *16*, 174–182.

Heinke, B., Ruscheweyh, R., Forsthuber, L., Wunderbaldinger, G., and Sandkühler, J. (2004). Physiological, neurochemical and morphological properties of a subgroup of GABAergic spinal lamina II neurones identified by expression of green fluorescent protein in mice. J. Physiol. *560*, 249–266.

Hughes, D.I., Sikander, S., Kinnon, C.M., Boyle, K.A., Watanabe, M., Callister, R.J., and Graham, B.A. (2012). Morphological, neurochemical and electrophysiological features of parvalbumin-expressing cells: a likely source of axo-axonic inputs in the mouse spinal dorsal horn. J. Physiol. *590*, 3927–3951.

Inan, S., and Cowan, A. (2004). Kappa opioid agonists suppress chloroquine-induced scratching in mice. Eur. J. Pharmacol. *502*, 233–237.

Iwagaki, N., Garzillo, F., Polgár, E., Riddell, J.S., and Todd, A.J. (2013). Neurochemical characterisation of lamina II inhibitory interneurons that express GFP in the PrP-GFP mouse. Mol. Pain *9*, 56.

Kivell, B., and Prisinzano, T.E. (2010). Kappa opioids and the modulation of pain. Psychopharmacology (Berl.) *210*, 109–119.

Kjellberg, F., and Tramèr, M.R. (2001). Pharmacological control of opioid-induced pruritus: a quantitative systematic review of randomized trials. Eur. J. Anaesthesiol. *18*, 346–357.

Ko, M.C., and Naughton, N.N. (2000). An experimental itch model in monkeys: characterization of intrathecal morphine-induced scratching and antinociception. Anesthesiology *92*, 795–805.

Ko, M.C., Lee, H., Song, M.S., Sobczyk-Kojiro, K., Mosberg, H.I., Kishioka, S., Woods, J.H., and Naughton, N.N. (2003). Activation of kappa-opioid receptors inhibits pruritus evoked by subcutaneous or intrathecal administration of morphine in monkeys. J. Pharmacol. Exp. Ther. *305*, 173–179.

König, M., Zimmer, A.M., Steiner, H., Holmes, P.V., Crawley, J.N., Brownstein, M.J., and Zimmer, A. (1996). Pain responses, anxiety and aggression in mice deficient in pre-proenkephalin. Nature *383*, 535–538.

Kumagai, H., Ebata, T., Takamori, K., Miyasato, K., Muramatsu, T., Nakamoto, H., Kurihara, M., Yanagita, T., and Suzuki, H. (2012). Efficacy and safety of a novel κ-agonist for managing intractable pruritus in dialysis patients. Am. J. Nephrol. *36*, 175–183.

Lagerström, M.C., Rogoz, K., Abrahamsen, B., Persson, E., Reinius, B., Nordenankar, K., Olund, C., Smith, C., Mendez, J.A., Chen, Z.F., et al. (2010). VGLUT2-dependent sensory neurons in the TRPV1 population regulate pain and itch. Neuron *68*, 529–542.

LaMotte, R.H., Shimada, S.G., and Sikand, P. (2011). Mouse models of acute, chemical itch and pain in humans. Exp. Dermatol. *20*, 778–782.

Leighton, G.E., Rodriguez, R.E., Hill, R.G., and Hughes, J. (1988). kappa-Opioid agonists produce antinociception after i.v. and i.c.v. but not intrathecal administration in the rat. Br. J. Pharmacol. *93*, 553–560.

Liao, C.C., Chang, C.S., Tseng, C.H., Sheen, M.J., Tsai, S.C., Chang, Y.L., and Wong, S.Y. (2011). Efficacy of intramuscular nalbuphine versus diphenhydramine for the prevention of epidural morphine-induced pruritus after cesarean delivery. Chang Gung Med. J. *34*, 172–178.

Liu, Y., Abdel Samad, O., Zhang, L., Duan, B., Tong, Q., Lopes, C., Ji, R.R., Lowell, B.B., and Ma, Q. (2010). VGLUT2-dependent glutamate release from nociceptors is required to sense pain and suppress itch. Neuron *68*, 543–556.

Loacker, S., Sayyah, M., Wittmann, W., Herzog, H., and Schwarzer, C. (2007). Endogenous dynorphin in epileptogenesis and epilepsy: anticonvulsant net effect via kappa opioid receptors. Brain *130*, 1017–1028.

Ma, Q. (2010). Labeled lines meet and talk: population coding of somatic sensations. J. Clin. Invest. *120*, 3773–3778.

Matthes, H.W., Maldonado, R., Simonin, F., Valverde, O., Slowe, S., Kitchen, I., Befort, K., Dierich, A., Le Meur, M., Dollé, P., et al. (1996). Loss of morphine-induced analgesia, reward effect and withdrawal symptoms in mice lacking the mu-opioid-receptor gene. Nature *383*, 819–823.

Maxwell, D.J., Belle, M.D., Cheunsuang, O., Stewart, A., and Morris, R. (2007). Morphology of inhibitory and excitatory interneurons in superficial laminae of the rat dorsal horn. J. Physiol. *584*, 521–533.

Mishra, S.K., and Hoon, M.A. (2013). The cells and circuitry for itch responses in mice. Science *340*, 968–971.

Miyamoto, T., Nojima, H., Shinkado, T., Nakahashi, T., and Kuraishi, Y. (2002). Itch-associated response induced by experimental dry skin in mice. Jpn. J. Pharmacol. *88*, 285–292.

Morgan, M.M., and Christie, M.J. (2011). Analysis of opioid efficacy, tolerance, addiction and dependence from cell culture to human. Br. J. Pharmacol. *164*, 1322–1334.

Nilsson, H.J., Levinsson, A., and Schouenborg, J. (1997). Cutaneous field stimulation (CFS): a new powerful method to combat itch. Pain *71*, 49–55.

Pan, Z.Z., Tershner, S.A., and Fields, H.L. (1997). Cellular mechanism for anti-analgesic action of agonists of the kappa-opioid receptor. Nature *389*, 382–385.

Patel, K.N., and Dong, X. (2010). An itch to be scratched. Neuron *68*, 334–339.

Peckys, D., and Landwehrmeyer, G.B. (1999). Expression of mu, kappa, and delta opioid receptor messenger RNA in the human CNS: a 33P in situ hybridization study. Neuroscience *88*, 1093–1135.

Pfeiffer, A., Brantl, V., Herz, A., and Emrich, H.M. (1986). Psychotomimesis mediated by kappa opiate receptors. Science *233*, 774–776.

Polgár, E., Hughes, D.I., Riddell, J.S., Maxwell, D.J., Puskár, Z., and Todd, A.J. (2003). Selective loss of spinal GABAergic or glycinergic neurons is not necessary for development of thermal hyperalgesia in the chronic constriction injury model of neuropathic pain. Pain *104*, 229–239.

Polgár, E., Gray, S., Riddell, J.S., and Todd, A.J. (2004). Lack of evidence for significant neuronal loss in laminae I-III of the spinal dorsal horn of the rat in the chronic constriction injury model. Pain *111*, 144–150.

Polgár, E., Durrieux, C., Hughes, D.I., and Todd, A.J. (2013a). A quantitative study of inhibitory interneurons in laminae I-III of the mouse spinal dorsal horn. PLoS ONE *8*, e78309.

Polgár, E., Sardella, T.C., Tiong, S.Y., Locke, S., Watanabe, M., and Todd, A.J. (2013b). Functional differences between neurochemically defined populations of inhibitory interneurons in the rat spinal dorsal horn. Pain *154*, 2606–2615.

Prescott, S.A., Ma, Q., and De Koninck, Y. (2014). Normal and abnormal coding of somatosensory stimuli causing pain. Nat. Neurosci. *17*, 183–191.

Roberson, D.P., Gudes, S., Sprague, J.M., Patoski, H.A., Robson, V.K., Blasl, F., Duan, B., Oh, S.B., Bean, B.P., Ma, Q., et al. (2013). Activity-dependent silencing reveals functionally distinct itch-generating sensory neurons. Nat. Neurosci. *16*, 910–918.

Ross, S.E. (2011). Pain and itch: insights into the neural circuits of aversive somatosensation in health and disease. Curr. Opin. Neurobiol. *21*, 880–887.

Ross, S.E., Mardinly, A.R., McCord, A.E., Zurawski, J., Cohen, S., Jung, C., Hu, L., Mok, S.I., Shah, A., Savner, E.M., et al. (2010). Loss of inhibitory interneurons in the dorsal spinal cord and elevated itch in Bhlhb5 mutant mice. Neuron *65*, 886–898.

Ross, S.E., McCord, A.E., Jung, C., Atan, D., Mok, S.I., Hemberg, M., Kim, T.K., Salogiannis, J., Hu, L., Cohen, S., et al. (2012). Bhlhb5 and Prdm8 form a repressor complex involved in neuronal circuit assembly. Neuron *73*, 292–303.

Ruscheweyh, R., and Sandkühler, J. (2002). Lamina-specific membrane and discharge properties of rat spinal dorsal horn neurones in vitro. J. Physiol. *541*, 231–244.

Sandkühler, J. (2009). Models and mechanisms of hyperalgesia and allodynia. Physiol. Rev. *89*, 707–758.

Sardella, T.C., Polgár, E., Garzillo, F., Furuta, T., Kaneko, T., Watanabe, M., and Todd, A.J. (2011). Dynorphin is expressed primarily by GABAergic neurons that contain galanin in the rat dorsal horn. Mol. Pain *7*, 76.

Schlaepfer, T.E., Strain, E.C., Greenberg, B.D., Preston, K.L., Lancaster, E., Bigelow, G.E., Barta, P.E., and Pearlson, G.D. (1998). Site of opioid action in the human brain: mu and kappa agonists' subjective and cerebral blood flow effects. Am. J. Psychiatry *155*, 470–473.

Seybold, V.S., Hylden, J.L., and Wilcox, G.L. (1982). Intrathecal substance P and somatostatin in rats: behaviors indicative of sensation. Peptides *3*, 49–54.

Shimada, S.G., and LaMotte, R.H. (2008). Behavioral differentiation between itch and pain in mouse. Pain *139*, 681–687.

Simmons, D.R., Spike, R.C., and Todd, A.J. (1995). Galanin is contained in GABAergic neurons in the rat spinal dorsal horn. Neurosci. Lett. *187*, 119–122.

Spike, R.C., Todd, A.J., and Johnston, H.M. (1993). Coexistence of NADPH diaphorase with GABA, glycine, and acetylcholine in rat spinal cord. J. Comp. Neurol. *335*, 320–333.

Stevens, C.W., and Yaksh, T.L. (1986). Dynorphin A and related peptides administered intrathecally in the rat: a search for putative kappa opiate receptor activity. J. Pharmacol. Exp. Ther. *238*, 833–838.

Sun, Y.G., Zhao, Z.Q., Meng, X.L., Yin, J., Liu, X.Y., and Chen, Z.F. (2009). Cellular basis of itch sensation. Science *325*, 1531–1534.

Szarvas, S., Harmon, D., and Murphy, D. (2003). Neuraxial opioid-induced pruritus: a review. J. Clin. Anesth. *15*, 234–239.

Tiong, S.Y., Polgár, E., van Kralingen, J.C., Watanabe, M., and Todd, A.J. (2011). Galanin-immunoreactivity identifies a distinct population of inhibitory interneurons in laminae I-III of the rat spinal cord. Mol. Pain *7*, 36.

Todd, A.J. (2010). Neuronal circuitry for pain processing in the dorsal horn. Nat. Rev. Neurosci. *11*, 823–836.

Todd, A.J., Spike, R.C., and Polgár, E. (1998). A quantitative study of neurons which express neurokinin-1 or somatostatin sst2a receptor in rat spinal dorsal horn. Neuroscience *85*, 459–473.

Togashi, Y., Umeuchi, H., Okano, K., Ando, N., Yoshizawa, Y., Honda, T., Kawamura, K., Endoh, T., Utsumi, J., Kamei, J., et al. (2002). Antipruritic activity of the kappa-opioid receptor agonist, TRK-820. Eur. J. Pharmacol. *435*, 259–264.

Vanderah, T.W. (2010). Delta and kappa opioid receptors as suitable drug targets for pain. Clin. J. Pain *26* (*Suppl 10*), S10–S15.

Ward, L., Wright, E., and McMahon, S.B. (1996). A comparison of the effects of noxious and innocuous counterstimuli on experimentally induced itch and pain. Pain *64*, 129–138.

Weisshaar, E., and Dalgard, F. (2009). Epidemiology of itch: adding to the burden of skin morbidity. Acta Derm. Venereol. *89*, 339–350.

Wikström, B., Gellert, R., Ladefoged, S.D., Danda, Y., Akai, M., Ide, K., Ogasawara, M., Kawashima, Y., Ueno, K., Mori, A., and Ueno, Y. (2005). Kappa-opioid system in uremic pruritus: multicenter, randomized, double-blind, placebo-controlled clinical studies. J. Am. Soc. Nephrol. *16*, 3742–3747.

Williams, J.T., Ingram, S.L., Henderson, G., Chavkin, C., von Zastrow, M., Schulz, S., Koch, T., Evans, C.J., and Christie, M.J. (2013). Regulation of μ-opioid receptors: desensitization, phosphorylation, internalization, and tolerance. Pharmacol. Rev. *65*, 223–254.

Xin, L., Geller, E.B., and Adler, M.W. (1997). Body temperature and analgesic effects of selective mu and kappa opioid receptor agonists microdialyzed into rat brain. J. Pharmacol. Exp. Ther. *281*, 499–507.

Yasaka, T., Kato, G., Furue, H., Rashid, M.H., Sonohata, M., Tamae, A., Murata, Y., Masuko, S., and Yoshimura, M. (2007). Cell-type-specific excitatory and inhibitory circuits involving primary afferents in the substantia gelatinosa of the rat spinal dorsal horn in vitro. J. Physiol. *581*, 603–618.

Yasaka, T., Tiong, S.Y., Hughes, D.I., Riddell, J.S., and Todd, A.J. (2010). Populations of inhibitory and excitatory interneurons in lamina II of the adult rat spinal dorsal horn revealed by a combined electrophysiological and anatomical approach. Pain *151*, 475–488.

Yosipovitch, G. (2008). Epidemiology of itching in skin and systemic diseases. In Itch: Basic Mechanisms and Therapy, G. Yosipovitch, M.W. Greaves, A.B. Fleischer, and F. McGlone, eds. (New York: Marcel Dekker), pp. 183–191.

Yosipovitch, G., Duque, M.I., Fast, K., Dawn, A.G., and Coghill, R.C. (2007). Scratching and noxious heat stimuli inhibit itch in humans: a psychophysical study. Br. J. Dermatol. *156*, 629–634.

Zheng, J., Lu, Y., and Perl, E.R. (2010). Inhibitory neurones of the spinal substantia gelatinosa mediate interaction of signals from primary afferents. J. Physiol. *588*, 2065–2075.

Neuron, Volume *82*

Supplemental Information

Dynorphin Acts as a Neuromodulator to Inhibit Itch in the Dorsal Horn of the Spinal Cord

Adam P. Kardon, Erika Polgár, Junichi Hachisuka, Lindsey M. Snyder, Darren Cameron, Sinead Savage, Xiaoyun Cai, Sergei Karnup, Christopher R. Fan, Gregory M. Hemenway, Carcha S. Bernard, Erica S. Schwartz, Hiroshi Nagase, Christoph Schwarzer, Masahiko Watanabe, Takahiro Furuta, Takeshi Kaneko, H. Richard Koerber, Andrew J. Todd, and Sarah E. Ross

Supplemental Information

Dynorphin is a neuromodulator that inhibits itch in the dorsal horn of the spinal cord

Adam P. Kardon, Erika Polgár, Junichi Hachisuka, Lindsey M. Snyder, Darren Cameron, Sinead Savage, Xiaoyun Cai, Sergei Karnup, Christopher R. Fan, Gregory M. Hemenway, Carcha S. Bernard, Erica S. Schwartz, Hiroshi Nagase, Christoph Schwarzer, Masahiko Watanabe, Takahiro Furuta, Takeshi Kaneko, H. Richard Koerber, Andrew J. Todd, and Sarah E. Ross

Supplemental Data

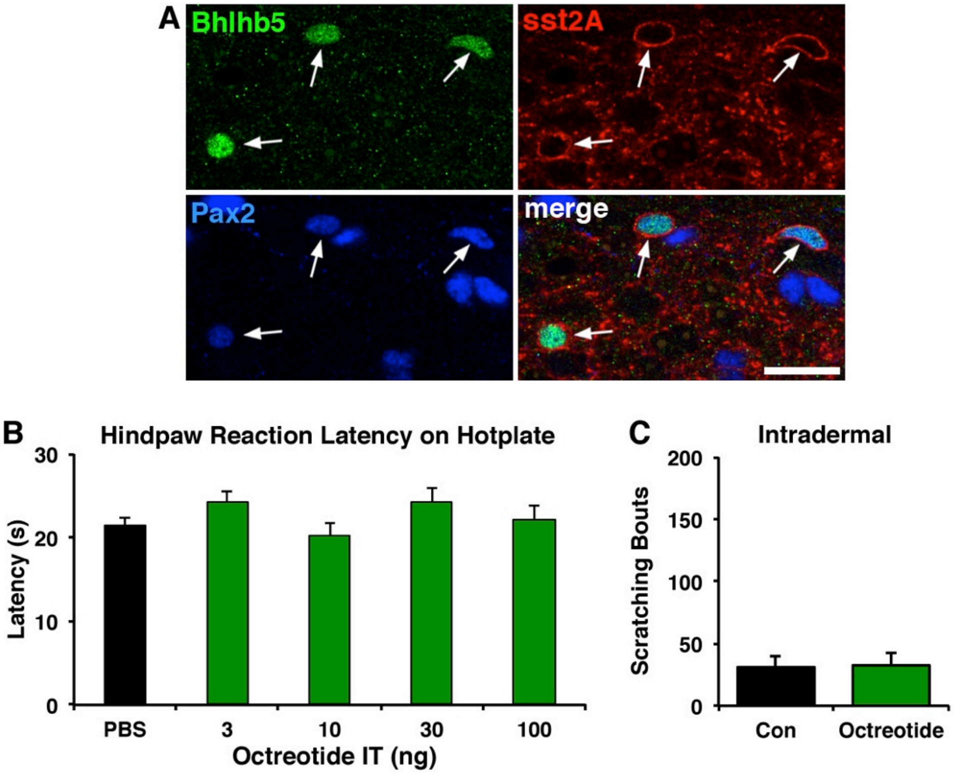

Figure S1, related to Figure 1. Analysis of sst_{2A}-expressing cells and the effect of the sst_{2A} agonist octreotide
A) B5-I neurons co-express sst_{2A} Bhlhb5-expressing neurons in the superficial dorsal horn that express Pax2 (B5-I neurons) also co-express sst_{2A}. Spinal cord sections from mice at post-natal day 4 (P4) were immunostained with antibodies directed against Bhlhb5 (green), sst_{2A} (red) and the inhibitory marker Pax2 (blue), as indicated. The vast majority (~90%) of cells expressing Bhlhb5 and Pax2 in laminae I and II co-label with sst_{2A} (arrows). A single confocal optical section of neurons in laminae I – IIo is shown. Scale bar = 20 µm**. B) Octreotide has no significant effect on acute thermal nociception when injected intrathecally** Response latency on a hotplate as assessed by lifting of the hindpaw or jumping following intrathecal injections of octreotide (3, 10, 30, or 100 ng) or vehicle (PBS). There was no significant difference between mice that received any dose of octreotide or vehicle. Two trials were averaged for each mouse (n = 6 – 8 mice/treatment). A one-way ANOVA was used to compare latency to react across treatments. **C) Octreotide does not elicit scratching behavior when injected intradermally.** Mice (n = 6 mice/treatment) received an intradermal injection of octreotide (100 ng) or vehicle (Con, 0.1X PBS) delivered into the nape of the neck, and were videotaped for forty minutes immediately following the injection. The number of scratch bouts by mice receiving intradermal octreotide was not significantly different than for those receiving vehicle alone. A Student's t-test was used to determine significance between treatment groups.

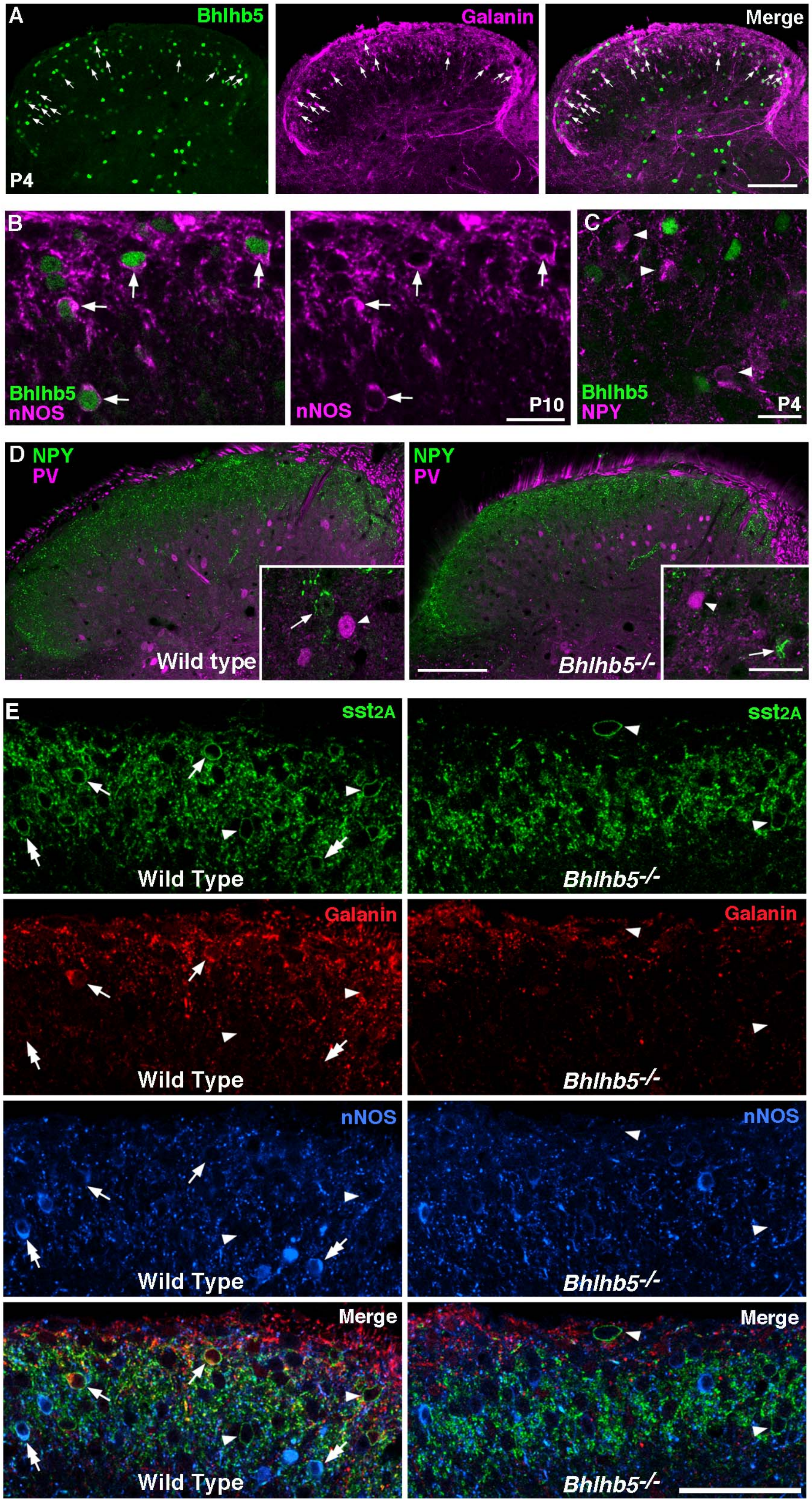

Figure S2, related to Figure 2. Immunohistochemical characterization of Bhlhb5-expressing interneurons in the dorsal horn of the spinal cord and comparison to *Bhlhb5* knockout mice

A) The majority of galanin-expressing neurons in the superficial dorsal horn of the spinal cord co-express Bhlhb5. Sections from P4 mice were immunostained with antibodies against Bhlhb5 (green) and galanin (magenta). A low magnification view (single confocal optical image) shows the entire dorsal horn. Merged view shows that virtually all galanin-positive neurons also express Bhlhb5 (arrows). Scale bar = 100 µm **B)** Large-field view of the section illustrated in Figure 2A from a P10 mouse reveals that many nNOS-expressing neurons are also Bhlhb5-immunoreative (arrows) Scale bar = 20 µm. **C)** Representative section from a P4 mouse immunostained for the presence of Bhlhb5 (green) and NPY (magenta) shows a lack of Bhlhb5 in NPY-expressing neurons (arrowheads). **D)** No change in the staining pattern for NPY or parvalbumin (PV) was observed in *Bhlhb5-/-* mice compared to wild type controls (sections from 6 mice examined for each genotype) Scale bar = 100 µm. Insets show individual NPY (arrows) and parvalbumin (arrowheads) cell bodies at higher magnification. Scale bar = 20µm. **E)** Larger field view of the section illustrated in Figure 2C, showing triple immunostaining with antibodies against sst_{2A} (green), galanin (red) and nNOS (blue) in 4 - 5 week old *Bhlhb5-/-* and wild type mice (n = 6 mice/genotype). Representative images demonstrate loss of the galanin- and nNOS-expressing subgroups (single- and double-headed arrows, respectively) of the sst_{2A}-immunoreactive population. The number of cells that express sst_{2A} but not galanin or nNOS (arrowheads) did not differ between *Bhlhb5-/-* and wild type mice. Scale bar = 50 µm.

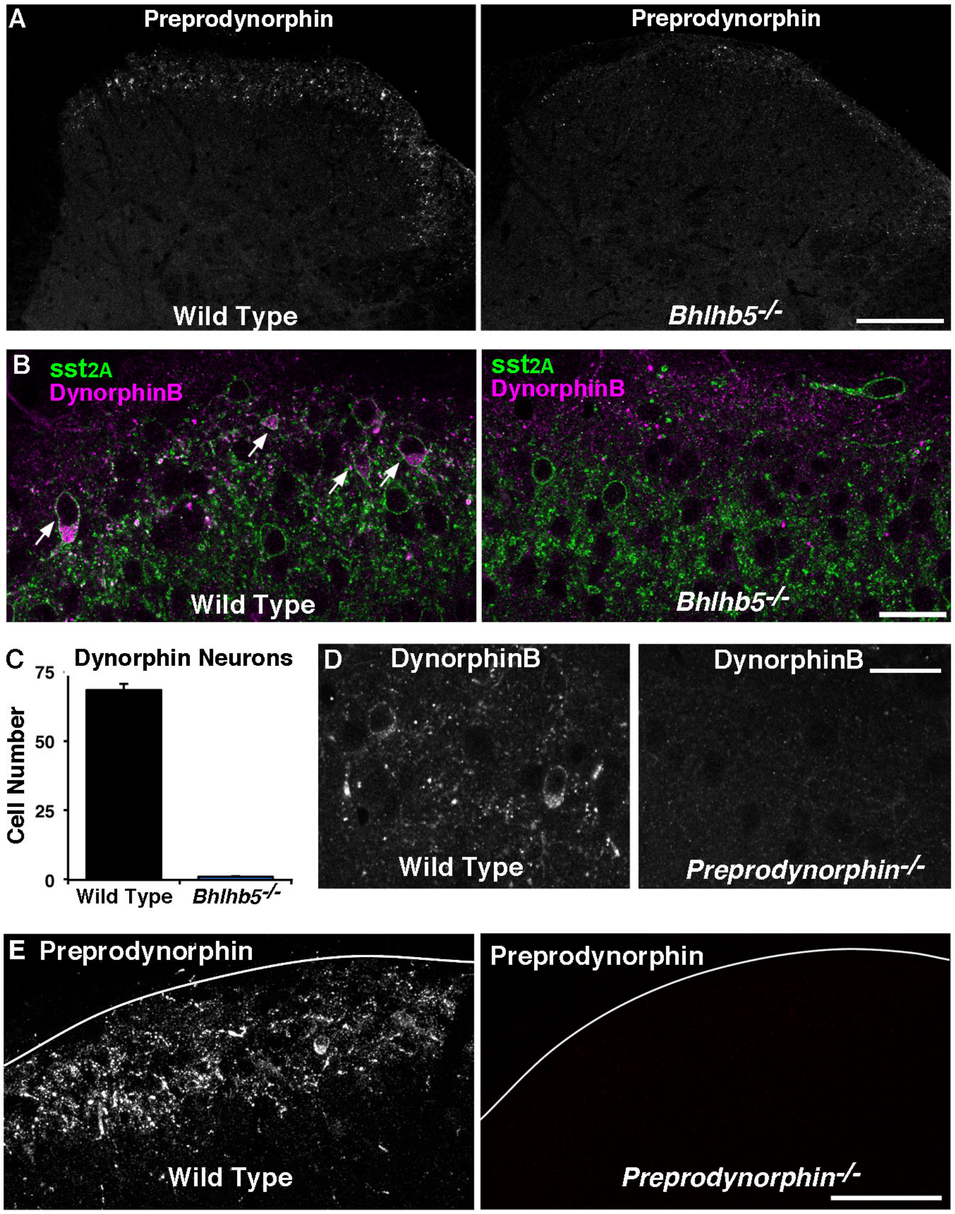

Figure S3, related to Figure 3. The number of dynorphin-expressing neurons is dramatically reduced in *Bhlhb5-/-* mice
A) Comparison of preprodynorphin immunoreactivity in 4 - 5 week old wild type and *Bhlhb5-/-* littermate pairs (representative confocal images, n = 3 mice/genotype). A low magnification view (single confocal optical image) reveals a dramatic loss of preprodynorphin-expressing neurons in laminae I-II. Scale bar = 100 µm. **B)** Similar results are obtained with an antibody against the dynorphin B peptide. Sections from wild type and *Bhlhb5-/-* mice were immunostained with antibodies directed against sst_{2A} (green) or dynorphin B (magenta). Dynorphin B-expressing sst_{2A}^{+} neurons in the wild type are indicated (arrows), but these are virtually absent in the *Bhlhb5-/-* mice. Scale bar = 20 µm. **C)** Quantification of (B). Data are mean + SEM number of cells in laminae I-II per dorsal horn through 100 µm cord taken from L4 (n = 3 mice/genotype). Note that the apparent number of dynorphin-expressing neurons as assessed with the dynorphin B antibody shown here is slightly lower than what is observed using the preprodynophin antibody (Figure 3C). This small difference is likely because the preprodynorphin antibody is more sensitive than the dynorphin B antibody, and can therefore detect cells that express very low levels of dynorphin. **D-E)** Immunostaining of tissue from wild type and *Preprodynorphin-/-* mice reveals that antibodies to dynorphin B (D) and preprodynorphin (E) are specific. Scale bar = 20 µm for D and 100 µm for E.

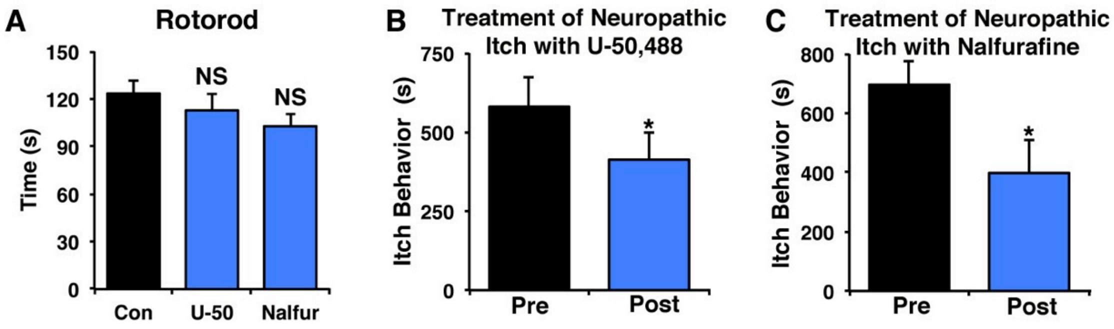

Figure S4, related to Figure 4. Effect of kappa agonists on motor function and neuropathic itch
A) No significant difference (NS, $p > 0.05$) in motor function is observed in a rotarod test upon treatment with U-50,488 (3 mg/kg) or nalfurafine (20 µg/kg) relative to control (PBS). Data are mean + SEM and analyzed using a one-way ANOVA ($n = 8$ mice/treatment). **B-C)** At 4 - 6 weeks of age, *Bhlhb5-/-* mice develop pruritic skin lesions, and can be considered a model of neuropathic itch. In order to assess the effect of kappa opioid receptor agonists on neuropathic itch, we measured the amount of time that *Bhlhb5-/-* mice with skin lesions spent biting/licking the affected site pre- and post-treatment. Mice with skin lesions spent the majority of the 30-minute observation period biting/licking the site of the lesion. However, following treatment with either (B) U-50,488 (3 mg/kg) or (C) nalfurafine (20 µg/kg), itch behavior is significantly reduced. Data are represented as mean + SEM ($n = 7$ - 11 mice/treatment). Statistical significance (*, $p < 0.05$) was determined by using a repeated measures t-test.

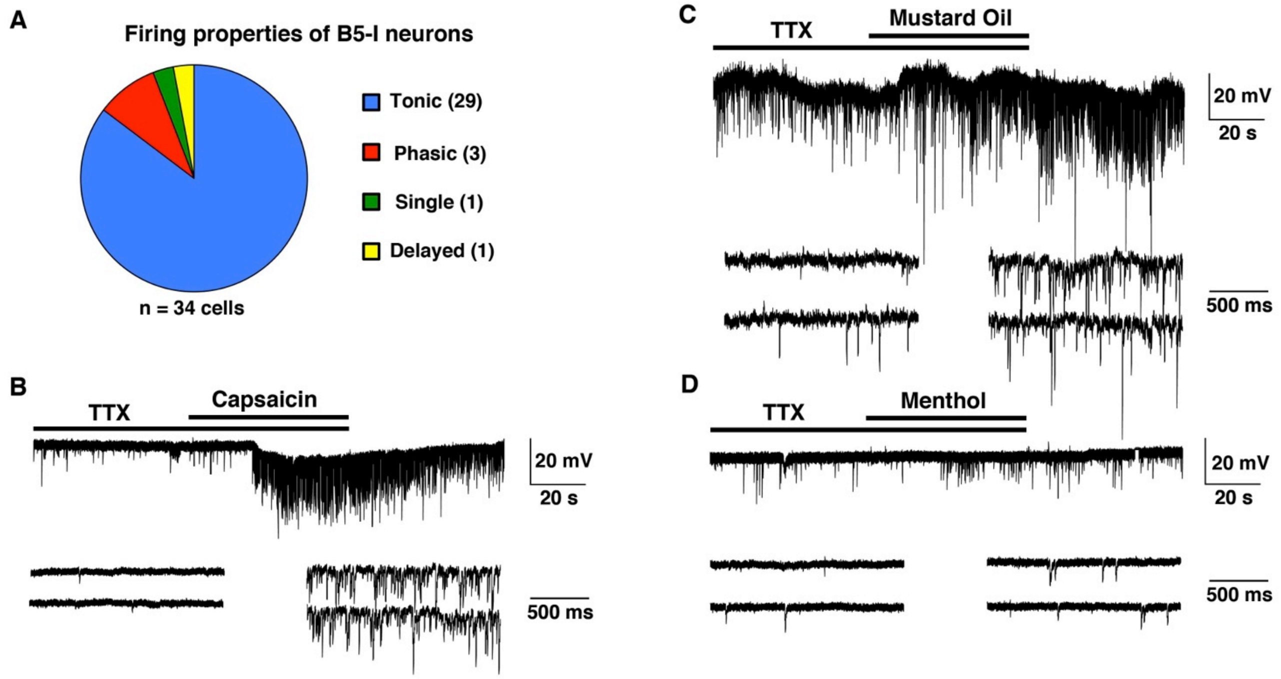

Figure S5, related to Figure 7. Electrophysiological analysis of B5-I neurons
A) The majority (29 of 34; 85%) of B5-I neurons showed tonic firing pattern upon injection of depolarising current. **B-D)** B5-I neurons receive direct input from TrpV1-, TrpA1- and TrpM8- expressing primary afferents. Increased mEPSC frequency following bath application of 2 µM capsaicin ($n = 3$, B), 100 µM AITC (mustard oil; $n = 5$, C) or 500 µM menthol ($n = 3$, D) was still observed in the presence of TTX (0.5 µM) to block action potential propagation, indicating that these effects are direct.

Supplemental Experimental Procedures:

Animal husbandry

Bhlhb5-/- mice were generated as previously described and are maintained on a mixed C57bl/6.129J background (Ross et al., 2010). To generate wild-type and *Bhlhb5-/-* mice for behavioral and immunohistochemical analyses, heterozygous *Bhlhb5-/+* mice were harem mated and age-matched wild-type and *Bhlhb5-/-* offspring from the resulting litters were used for subsequent experiments. Unless otherwise stated, behavioral experiments that involved *Bhlhb5-/-* mice were performed on 4 – 5 week old mice that did not have skin lesions. For electrophysiological experiments, *Bhlhb5-cre* mice, described in (Ross et al., 2010) were mated with Ai9 cre-responsive tdTomato reporter mice (The Jackson Laboratory; strain 007905). Mice were given free access to food and water and housed under standard laboratory conditions. The use of animals was approved by the Institutional Animal Care and Use Committee of the University of Pittsburgh and/or the Ethical Review Process Applications Panel of the University of Glasgow. Experiments performed in A.J.T.'s lab were in accordance with the UK Animals (Scientific Procedures) Act 1986.

Immunohistochemistry

Young adult or neonatal mice were deeply anaesthetized and fixed by perfusion with 4% freshly depolymerized formaldehyde. Transverse 60 µm thick sections were cut with a vibrating microtome from mid-lumbar spinal cord segments (L3 or L4) and processed free-floating for immunocytochemistry. Details of the primary antibodies used in this study are given in Supplementary Table 1. These were revealed with species-specific secondary antibodies raised in donkey and conjugated to Pacific Blue or Alexa 488 (both from Life Technologies) or to Rhodamine Red or DyLight 649 (both from Jackson Immunoresearch). In some cases tyramide signal amplification (TSA) was used. Sections were scanned with a confocal microscope (Zeiss LSM 710, with Argon multi-line, 405 nm diode, 561 nm solid state and 633 nm HeNe lasers lasers, and spectral detection system) through a 40× oil-immersion lens (NA 1.3) with the pinhole set to 1 Airy unit. Scans were analyzed with Neurolucida for Confocal (Microbrightfield). All quantitative analyses were carried out on the superficial dorsal horn (laminae I and II). In all cases involving comparison of knockout and wild-type mice, the observer was blind to the genotype.

Antibody characterization

The Bhlhb5 antibody was generated against the N-terminal part of the mouse protein, and detects a band at 37 kDa on Western blots of wild-type mouse brain that is absent in extracts from Bhlhb5 knockouts (Ross et al., 2010). The mouse monoclonal antibody NeuN, raised against cell nuclei from mouse brain, reacts with a neuron-specific protein (Mullen et al., 1992). In rat spinal cord, NeuN apparently detects all neurons, but does not label glial cells (Todd et al., 1998). Staining with the galanin antibody is absent from brains of galanin knockout mice (Makwana et al., 2010), and we have shown that staining in the dorsal horn with both galanin and NPY antibodies is abolished by preabsorption with the corresponding peptide (Rowan et al., 1993; Simmons et al., 1995). The two PPD antibodies were raised against the C-terminal 20 amino acids (FKVVTRSQENPNTYSEDLDV) of rat PPD (Lee et al., 1997), while the dynorphin B antibody was against the full peptide (YGGFLRRQFKVVT), which shows limited overlap with the sequence used to raise the PPD antibodies (Griffond et al., 1993). Staining with all 3 antibodies was absent in spinal cord sections from PPD knockout mice (Loacker et al., 2007). The Pax2 antibody was raised against amino acids 188-385 of the mouse protein, and recognizes bands of the appropriate size on Western blots of mouse embryonic kidney (Dressler and Douglass, 1992). The nNOS antibody was raised against recombinant rat protein and detects a band of 155 kDa in extracts of rat hypothalamus (Herbison et al., 1996). The sst_{2A} antibody was generated against the C terminal 15 amino acids of the mouse receptor, and staining is abolished by incubation with the immunizing peptide (manufacturer's specification). The parvalbumin antibody was raised against the mouse protein and recognizes a band at 13 kDa on Western blots of mouse brain (Nakamura et al., 2004).

Expression of neurochemical markers in wild type and Bhlhb5-/- mice

Sections from P4 or P10 C57bl/6J mice (University of Glasgow, Biological Services) were reacted with antibodies against Bhlhb5 and either anti-NPY, anti-galanin, or anti nNOS. Six *Bhlhb5-/-* and wild-type littermate pairs (4-5 week old) were used to determine the proportion of neurons that expressed sst_{2A}, galanin or nNOS. Sections were reacted with antibodies against each of these. Confocal image stacks (1 µm z-separation) were obtained from one dorsal horn from each animal and analyzed using a modification of the optical dissector method (Polgár et al., 2004). To examine the distribution of NPY and parvalbumin, 2 sections from each mouse were reacted with the corresponding antibodies and scanned with the confocal microscope. To look for alterations in the number of dynorphin-expressing cells, sections were reacted with sst_{2A} and either anti-PPD or anti-dynorphin B. One dorsal horn from each of 3 wild type and 3 *Bhlhb5* knockout mice were analyzed for each antibody combination. In order to ensure specificity of the PPD and dynorphinB antibodies, sections from wild-type and *preprodynorphin-/-* mice (Loacker et al., 2007) were reacted with these antibodies.

Identification of B5-I neurons in neonatal mice

Sections from three P4 C57bl/6J mice (University of Glasgow Biological Services) were reacted with cocktails of antibodies against Bhlhb5, NeuN, NPY, galanin, PPD, and/or Pax2, as indicated. To estimate the proportion of Bhlhb5-immunoreactive neurons in superficial dorsal horn that were inhibitory ($Pax2^+$), all $Bhlhb5^+$/$NeuN^+$ profiles in the dorsal horn on one side from each of two sections per animal were identified and plotted. These were then examined to determine whether they were Pax2-immunoreactive. Expression of galanin, NPY and PPD in $Bhlhb5^+$ neurons were assessed by determining the proportion of the neurons immunoreactive for each peptide that were also Bhlhb5-immunoreactive. In the case of galanin and PPD, we also

determined the proportion of Bhlhb5-expressing inhibitory interneurons (Bhlhb5^{+}/Pax2^{+} cells) that were immunoreactive for these peptides. Since there was relatively little expression of nNOS in laminae I-II at P4, we reacted sections from 3 P10 mice with antibodies against nNOS, Pax2 and Bhlhb5. Confocal scans were examined to test whether any nNOS-expressing inhibitory interneurons (nNOS^{+}/Pax2^{+} cells) were Bhlhb5-immunoreactive.

Table S1. Primary antibodies used in this study

Antibody	Host	Supplier/reference	Catalog number	Dilution
Bhlhb5	Rat	Ross et al., 2010		1:1,000
NeuN	Mouse	Millipore	MAB377	1:500-1,000
Galanin	Rabbit	Bachem	T-4334	1:20,000 (TSA)<br>1:1,000
PPD	Guinea pig	Lee et al., 1997		1:5,000 (TSA)
PPD	Rabbit	Lee et al., 1997		1:10,000 (TSA)
NPY	Rabbit	Bachem	T-4070	1:1,000
Pax2	Rabbit	Life technologies	716000	1:1,000
nNOS	Sheep	Herbison et al., 1996		1:2,000
sst_{2A}	Guinea pig	Gramsch laboratories	SS-870	1:2,000
parvalbumin	Guinea pig	Nakamura et al., 2004		1:2,500
Dynorphin B	Rabbit	Griffond et al., 1993		1:1,000

TSA: detected with tyramide signal amplification

Expression of neurochemical markers in wild-type and Bhlhb5-/- mice

Six (4- to 5-week old) mice of each genotype were used to determine the proportion of neurons that expressed sst_{2A}, galanin or nNOS. Sections were reacted with antibodies against each of these, together with NeuN antibody. Following incubation with fluorescent secondary antibodies, cell nuclei were stained with Sytox Orange (Life Technologies). Confocal image stacks (1 µm z-separation) were obtained from one dorsal horn from each animal and analyzed using a modification of the optical disector method (Polgár et al., 2004). The 5th and 40th optical sections in the z-series were set as reference and look-up, and all sections between these two were examined. The color channels representing NeuN and Sytox Orange were initially switched on, and all neuronal nuclei that were present on the reference section or any of the subsequent sections in the series, and that had disappeared by the look-up section, were drawn onto an outline of the gray matter. The channels representing sst_{2A}, galanin and nNOS were then viewed and the presence or absence of each type of immunoreactivity was noted for each neuron in the disector sample. Since sections can undergo a variable degree of shrinkage during processing, we corrected for this as described previously (Polgár et al. 2005). Briefly, the thickness of the mounted section was determined by scanning from the top to the bottom surface, and this was divided by 60 µm (the initial thickness) to provide a correction factor.

Since we found that all sst_{2A}-expressing cells remained Pax2-immunoreactive in adult wild-type mice, and that around 50% of all Pax2-expressing neurons in these animals were sst_{2A}-immunoreactive, we concluded that Pax2 can be used as a marker for most (if not all) inhibitory interneurons in the superficial dorsal horn in the adult. In order to determine whether there was loss among inhibitory interneurons that lacked sst_{2A}, we performed immunofluorescence labeling on sections from wild-type and *Bhlhb5-/-* mice with sst_{2A}, Pax2 and NeuN antibodies and then stained nuclei with DAPI (Sigma). Confocal image stacks (1 µm z-separation) were scanned through 2 dorsal horns from each mouse. These were analyzed by using the optical disector method, with a 10 µm separation between reference and look-up sections, and the number of Pax2^{+} cells with or without sst_{2A} immunoreactivity was determined. Again, a correction was made for tissue shrinkage. To examine the distribution of NPY and parvalbumin, 2 sections from each mouse were reacted with the corresponding antibodies and scanned with the confocal microscope. To look for alterations in the number of dynorphin-expressing cells, sections were reacted with sst_{2A} and either anti-PPD or anti-dynorphin B. One dorsal horn from each of 3 wild-type and 3 *Bhlhb5* knockout mice were analyzed for each antibody combination. The sections were scanned through their full thickness (1 µm z-separation) and the number of sst_{2A}-immunoreactive cells that were either PPD- or dynorphin B-positive was determined.

Behavioral assessment

Unless otherwise noted, 6 - 8 week-old male C57bl/6J mice were used for all behavioral tests. The injection sites were shaved at least twenty-four hours prior to the start of an experiment. Mice were placed in clear plastic containers (3.5” x 3.5” x 5”) for observation and allowed to acclimate for 30 minutes prior to all behavioral assessments. All assays were videotaped, and subsequently scored by an experimenter blind to treatment and/or genotype.

Intrathecal injections

A modified version (Lee et al., 2007) of the original intrathecal injection method (Hylden and Wilcox, 1980) was used. Mice were anesthetized (2.0% for induction and 1.5% for maintenance) with isoflurane in a flow of O_2, placed in a prone position, and the hair on their back was clipped. The caudal paralumbar region, just cranial to the iliac crests, was securely held by the thumb and middle fingers of the left hand, and the index finger was placed on the tip of sixth lumbar (L6) spinous process, the highest point of the vertebral column. All intrathecal injections were delivered in a total volume of 5 µl using a 30-gauge needle attached to a luer-tip 25 µl Hamilton syringe. The needle was inserted into the tissue at a 45° angle. The angle of the needle was maintained until the needle went through the fifth intervertebral space (L5–L6) and “slipped in” causing a sudden lateral movement of the tail. Solution was injected at a rate of 1 µl/s. The needle was held in position for 10 s and removed slowly to avoid any outflow of the solution. Anesthesia was discontinued and the mice recovered from anesthesia within 5 min.

Octreotide-evoked behavior

Mice were injected intrathecally with octreotide (3, 10, 30 or 100 ng) or vehicle (PBS), as described above. Immediately after recovering from the injection, mice were videotaped for 30 minutes to assess spontaneous scratching behavior. In some instances, chloroquine (100 µg in 10 µl PBS) was then injected intradermally into the calf of octreotide- and vehicle-treated mice. Itch behavior was defined as the cumulative amount of time spent biting/licking the injection site.

Acute itch behavior

5-HT (30 µg), histamine (100 µg), chloroquine (200 µg) and SLIGRL-NH2 (100 nM) were all dissolved in PBS and injected intradermally in a total volume of 20 µl into the nape of the neck. Each pruritogen evoked a robust scratching response that lasted approximately 30 minutes. Mice were pretreated systemically with U-50,488 (3 mg/kg; IP), nalfurafine (20 µg/kg; IP) or vehicle (50 µl PBS; IP) 30 minutes prior to the intradermal injections of pruritogens. The number of scratch bouts was counted in five-minute intervals over a forty-minute observation period.

AEW itch To model dry skin-mediated itch, spontaneous scratching behavior was induced by treatment twice daily with an acetone/diethylether (1:1) solution for 15 seconds followed by water for 30 seconds (AEW). Following five days of AEW treatment, mice received U-50,488, nalfurafine or vehicle and were videotaped for one hour. Scratch bouts directed to the nape of the neck and rostral back were counted in five-minute intervals.

Motor coordination

Mice were trained on the rotarod at a constant speed of 16 rpm until they could remain on for one minute without falling. On test day, mice were pretreated intraperitoneally with either U-50,488 (3mg/kg), nalfurafine (20µg/kg) or PBS. Each mouse performed three trials on the rotarod, which was accelerated from 4 rpm to 40 rpm over a period of five minutes. Trials were separated by 5-minute intervals for rest, and the time to fall off the apparatus was averaged across the trials for each mouse.

Cheek model of itch

For the cheek model of itch (Shimada and LaMotte, 2008), mice received an intradermal injection of either chloroquine (100 µg in 10 µl of PBS) or capsaicin (10 µl of 1% capsaicin, dissolved in a saline solution containing 7% Tween 80 and 20% ethanol) into the skin of the cheek. Observation chambers were surrounded by four mirrors such that the experimenter had an unobstructed view of the mouse’s cheek. Scratch bouts with the hindlimb and instances of forelimb wiping were videotaped and quantified over a 40-minute observation period.

Calf model of itch

In some experiments, mice were pretreated with nalfurafine (40 ng), U-50,488 (10 µg) or vehicle (PBS) delivered intrathecally 30 min in advance. In other experiments, mice were pretreated with kappa antagonists 5’GNTI (1 µg), nor-BNI (1 µg) or vehicle, delivered intrathecally 24 h in advance, based on Munro et al., (2012). At the time of the experiment, chloroquine (100 µg in 10 µl PBS) was injected intradermally in the front of the calf, and the response was videotaped for 60 minutes. During real-time playback, biting is difficult to distinguish from other behaviors such as licking or grooming with the forepaws. However, when played at ¼ speed, biting could be identified as a gnawing motion in which the head moves at a frequency of approximately 15 Hz interspersed with abrupt head jerks, whereas licking was observed as a slow bobbing of the head at a frequency of approximately 4 Hz (LaMotte et al., 2011).

Inhibition of itch by menthol

Menthol (8%; Stopain, Troy Healthcare) or PBS (control) was applied topically in a volume of 10 µl to the cheek. Application of menthol resulted in modest wiping behavior in both wild-type and *Bhlhb5-/-* mice that ceased within ~ 5 min. Ten minutes after

the initial application of menthol or PBS, mice were given an intradermal injection of chloroquine (100 µg in 10 µl) into the cheek. Mice were videotaped and scratch bouts with the hindlimb were quantified over a 30-minute observation period.

Hotplate
Mice were lightly anesthetized with isoflurane, and injected intrathecally with octreotide (3, 10, 30 or 100 ng) or vehicle (PBS) in a total volume of 5 µl. One hour later, mice were placed directly on a hot plate set to 55°C. The response latency to hind paw licking or jumping was recorded over two trials for each mouse. Mice that did not respond at 45 seconds were removed from the hot plate to avoid tissue damage.

Electrophysiology
Four- to six-week old mice were deeply anesthetized with urethane (1.2 – 1.5 g/kg, i.p.). Thoracolumbar laminectomy was performed, and the thoracic and lumbar spinal cord was excised and placed it into an ice-cold, sucrose-based Krebs solution equilibrated with 95% O_2/5% CO_2. The composition of sucrose-based Krebs solution was as follows (mM); 234 sucrose, 2.5 KCl, 0.5 $CaCl_2$, 10 $MgSO_4$, 1.25 NaH_2PO_4, 26 $NaHCO_3$, 11 Glucose. Immediately after the removal of the spinal cord, the mice were killed by exsanguination. Dura and pia-arachnoid membrane were removed after cutting all of the ventral and dorsal roots. The spinal cord was mounted on a vibratome and parasagittal or transverse slices (250-300 µm thickness) were made. The slices were incubated in oxygenated, sucrose-based Krebs solution for at least 30 min in room temperature before recording. The slice was then transferred to a recording chamber. The slice was fixed on a nylon harp and perfused with Krebs solution saturated with 95% O_2 and 5% CO_2 at 36 ± 1 °C at flow rate of 10 ml/min. The Krebs solution contained (mM): 117 NaCl, 3.6 KCl, 2.5 $CaCl_2$, 1.2 $MgCl_2$, 1.2 NaH_2PO_4, 25 $NaHCO_3$ and 11 glucose.

Lamina II was identified as a translucent band in the dorsal horn. Individual neurons were identified with a 40x objective lens under IR-DIC optics (BX51WI Olympus microscope). The microscope was coupled with a CCD camera (ORCA-ER Hamamatsu Photonics) and monitor screen. Genetically labeled *Bhlhb5-cre* neurons in lamina II of the dorsal horn were identified by their fluorescence. B5-I neurons were distinguished within this subset by hyperpolarization upon bath application of 1 µM somatostatin. To determine firing pattern of B5-I neurons, 1 s depolarizing current steps (20 – 200 pA in 10 pA steps) were applied. Because firing pattern can vary depending on holding potential, we tested the B5-I neurons at several different holding potentials. Firing patterns were determined in response to depolarizing current injections of 1 s duration from each of the potentials. Classifications were based on (Heinke et al., 2004; Ruscheweyh et al., 2004; Ruscheweyh and Sandkuhler, 2002).

Whole-cell patch-clamp recordings were made with patch-pipette electrodes with a resistance of 6-12 MΩ. The composition of the pipette solution was as follows (mM); 135 potassium gluconate, 5 KCl, 0.5 $CaCl_2$, 5 EGTA, 5 Hepes, 5 ATP-Mg, pH 7.2. Neurobiotin 0.2% or Alexa Fluor 488 25 µM were added for morphological experiments. The signals were acquired with an amplifier (Axopatch 200B, Molecular Devices, California). The data were digitized with an A/D converter (Digidata 1322A, Molecular Devices) and stored on a personal computer using a data acquisition program (Clampex version 9.0, Molecular Devices).

Cell recordings were made in voltage-clamp mode at holding potentials of -70mV to record excitatory postsynaptic currents (EPSCs). At this potential GABA-/ glycine-mediated inhibitory postsynaptic currents were negligible (Yoshimura and Nishi, 1993). Frequency and amplitude of EPSCs were analysed by using MiniAnalysis program (Synaptosoft, Inc.). We defined neurons as being sensitive to a particular drug when the frequency or amplitude of the synaptic responses was altered by more than ± 50 % of control. The drugs were dissolved in Krebs solution and applied by exchanging solutions via a three-way stopcock. The drugs used were somatostatin (1 µM, Sigma-Aldrich), capsaicin (2 *µ*M; Sigma-Aldrich), allyl isothiocyanate (100 µM; Sigma-Aldrich), menthol (500 µM; Sigma-Aldrich) and TTX (0.5 µM, Tocris).

Morphological reconstructions and characterization
Biocytin-filled cells were visualized as described previously (Karnup and Stelzer, 1999). Briefly, fixed slices were embedded in 10% gelatin and sectioned at 100 µm thickness using a vibratome (Leica Microsystems, Wetzlar, Germany). The sections were reacted with 1% H_2O_2, 0.5% Triton X-100, ABC complex, and Ni-DAB chromagen. After dehydration, the sections were mounted in DPX. Two-dimensional reconstructions of filled neurons were made with Neurolucida software (MicroBrightField, Colchester, VT).

Following reconstruction, neurons were classified into one of 5 major categories as previously identified (Grudt and Perl, 2002; Yasaka et al., 2007; Zheng et al., 2010) in the hamster, rat, and mouse, respectively. According to this scheme, islet cells display an elongated dentritic tree in the rostral-caudal dimension greater than 400 µm and a more limited dorsal-ventral and medial-lateral spread (<100 µm). The arborization of islet cells is mostly restricted to lamina II. Central cells show similar morphological characteristics to islet cells; however, the extent of the dendritic arbor in the rostral-caudal dimension is much less (~200 µm). Vertical cells show a greater dorsal-ventral spread of the dentritic tree into lamina III and sometimes lamina I (~200 µm). Radial cells display dendrites that spread in all directions at approximately equal lengths (between 50-150 µm) across all dimensions and remained mostly within lamina II. Cells not matching any of the above mentioned categories are designated as unclassified.

Supplemental References

Dressler, G.R., and Douglass, E.C. (1992). Pax-2 is a DNA-binding protein expressed in embryonic kidney and Wilms tumor. Proceedings of the National Academy of Sciences U S A *89*, 1179-1183.

Griffond, B., Deray, A., Fellmann, D., Ciofi, P., Croix, D., and Bugnon, C. (1993). Colocalization of prolactin- and dynorphin-like substances in a neuronal population of the rat lateral hypothalamus. Neuroscience Letters *156*, 91-95.

Grudt, T.J., and Perl, E.R. (2002). Correlations between neuronal morphology and electrophysiological features in the rodent superficial dorsal horn. The Journal of physiology *540*, 189-207.

Heinke, B., Ruscheweyh, R., Forsthuber, L., Wunderbaldinger, G., and Sandkuhler, J. (2004). Physiological, neurochemical and morphological properties of a subgroup of GABAergic spinal lamina II neurones identified by expression of green fluorescent protein in mice. The Journal of physiology *560*, 249-266.

Herbison, A.E., Simonian, S.X., Norris, P.J., and Emson, P.C. (1996). Relationship of neuronal nitric oxide synthase immunoreactivity to GnRH neurons in the ovariectomized and intact female rat. Journal of Neuroendocrinology *8*, 73-82.

Hylden, J.L., and Wilcox, G.L. (1980). Intrathecal morphine in mice: a new technique. European Journal of Pharmacology *67*, 313-316.

Karnup, S., and Stelzer, A. (1999). Temporal overlap of excitatory and inhibitory afferent input in guinea-pig CA1 pyramidal cells. The Journal of physiology *516 (Pt 2)*, 485-504.

LaMotte, R.H., Shimada, S.G., and Sikand, P. (2011). Mouse models of acute, chemical itch and pain in humans. Experimental dermatology *20*, 778-782.

Lee, T., Kaneko, T., Taki, K., and Mizuno, N. (1997). Preprodynorphin-, preproenkephalin-, and preprotachykinin-expressing neurons in the rat neostriatum: an analysis by immunocytochemistry and retrograde tracing. Journal of Comparative Neurology *386*, 229-244.

Lee, H., Naughton, N.N., Woods, J.H., and Ko, M.C. (2007). Effects of butorphanol on morphine-induced itch and analgesia in primates. Anesthesiology *107*, 478-485.

Loacker, S., Sayyah, M., Wittmann, W., Herzog, H., and Schwarzer, C. (2007). Endogenous dynorphin in epileptogenesis and epilepsy: anticonvulsant net effect via kappa opioid receptors. Brain : a journal of neurology *130*, 1017-1028.

Makwana, M., Werner, A., Acosta-Saltos, A., Gonitel, R., Pararajasingham, A., Ruff, C., Rumajogee, P., Cuthill, D., Galiano, M., Bohatschek, M.*, et al.* (2010). Peripheral facial nerve axotomy in mice causes sprouting of motor axons into perineuronal central white matter: time course and molecular characterization. Journal of Comparative Neurology *518*, 699-721.

Munro, T.A., Berry, L.M., Van't Veer, A., Beguin, C., Carroll, F.I., Zhao, Z., Carlezon, W.A., Jr., and Cohen, B.M. (2012). Long-acting kappa opioid antagonists nor-BNI, GNTI and JDTic: pharmacokinetics in mice and lipophilicity. BMC Pharmacology *12*, 5.

Mullen, R.J., Buck, C.R., and Smith, A.M. (1992). NeuN, a neuronal specific nuclear protein in vertebrates. Development *116*, 201-211.

Nakamura, M., Sato, K., Fukaya, M., Araishi, K., Aiba, A., Kano, M., and Watanabe, M. (2004). Signaling complex formation of phospholipase Cbeta4 with metabotropic glutamate receptor type 1alpha and 1,4,5-trisphosphate receptor at the perisynapse and endoplasmic reticulum in the mouse brain. The European journal of neuroscience *20*, 2929-2944.

Polgár, E., Gray, S., Riddell, J.S., and Todd, A.J. (2004). Lack of evidence for significant neuronal loss in laminae I-III of the spinal dorsal horn of the rat in the chronic constriction injury model. Pain *111*, 144-150.

Polgar, E., Hughes, D.I., Arham, A.Z., and Todd, A.J. (2005). Loss of neurons from laminas I-III of the spinal dorsal horn is not required for development of tactile allodynia in the spared nerve injury model of neuropathic pain. The Journal of neuroscience : the official journal of the Society for Neuroscience *25*, 6658-6666.

Ross, S.E., Mardinly, A.R., McCord, A.E., Zurawski, J., Cohen, S., Jung, C., Hu, L., Mok, S.I., Shah, A., Savner, E.M.*, et al.* (2010). Loss of inhibitory interneurons in the dorsal spinal cord and elevated itch in Bhlhb5 mutant mice. Neuron *65*, 886-898.

Rowan, S., Todd, A.J., and Spike, R.C. (1993). Evidence that neuropeptide Y is present in GABAergic neurons in the superficial dorsal horn of the rat spinal cord. Neuroscience *53*, 537-545.

Ruscheweyh, R., Ikeda, H., Heinke, B., and Sandkuhler, J. (2004). Distinctive membrane and discharge properties of rat spinal lamina I projection neurones in vitro. The Journal of physiology *555*, 527-543.

Ruscheweyh, R., and Sandkuhler, J. (2002). Lamina-specific membrane and discharge properties of rat spinal dorsal horn neurones in vitro. The Journal of physiology *541*, 231-244.

Shimada, S.G., and LaMotte, R.H. (2008). Behavioral differentiation between itch and pain in mouse. Pain *139*, 681-687.

Simmons, D.R., Spike, R.C., and Todd, A.J. (1995). Galanin is contained in GABAergic neurons in the rat spinal dorsal horn. Neuroscience Letters *187*, 119-122.

Todd, A.J., Spike, R.C., and Polgar, E. (1998). A quantitative study of neurons which express neurokinin-1 or somatostatin sst2a receptor in rat spinal dorsal horn. Neuroscience *85*, 459-473.

Yasaka, T., Kato, G., Furue, H., Rashid, M.H., Sonohata, M., Tamae, A., Murata, Y., Masuko, S., and Yoshimura, M. (2007). Cell-type-specific excitatory and inhibitory circuits involving primary afferents in the substantia gelatinosa of the rat spinal dorsal horn in vitro. The Journal of physiology *581*, 603-618.

Yoshimura, M., and Nishi, S. (1993). Blind patch-clamp recordings from substantia gelatinosa neurons in adult rat spinal cord slices: pharmacological properties of synaptic currents. Neuroscience *53*, 519-526.

Zheng, J., Lu, Y., and Perl, E.R. (2010). Inhibitory neurones of the spinal substantia gelatinosa mediate interaction of signals from primary afferents. The Journal of physiology *588*, 2065-2075.
